# Neuroimaging-informed phenotypes of suicidal behavior: a family history of suicide and the use of a violent suicidal means

**DOI:** 10.1038/s41398-018-0170-2

**Published:** 2018-06-19

**Authors:** Fabrice Jollant, Gerd Wagner, Stéphane Richard-Devantoy, Stefanie Köhler, Karl-Jürgen Bär, Gustavo Turecki, Fabricio Pereira

**Affiliations:** 1 McGill Group for Suicide Studies (MGSS), McGill University & Douglas Mental Health University Institute, Montréal, Canada; 2 Department of Psychiatry, Academic Hospital (CHU) of Nîmes, Nîmes, France; 3 Paris Descartes University & Sainte-Anne Hospital, Paris, France; 4 Department of Psychiatry and Psychotherapy, Jena University Hospital, Jena, Germany; 5 Department of Radiology, Academic Hospital (CHU) of Nîmes & Research Team EA2415, Nîmes, France

## Abstract

The identification of brain markers of suicidal risk is highly expected. However, neuroimaging studies have yielded mixed results, possibly due to phenotypic heterogeneity. In the present study, we addressed this issue using structural brain imaging. First, two independent samples of suicide attempters (*n* = 17 in Montreal, 32 in Jena), patient controls (*n* = 26/34), and healthy controls (*n* = 66/34) were scanned with magnetic resonance imaging. Groups were compared with FSL. We then reviewed the literature and run a GingerALE meta-analysis of 12 structural imaging studies comparing suicide attempters and patient controls with whole-brain analyses (*n* = 693). Finally, we explored the potential contribution of two variables previously associated with biological/cognitive deficits: a family history of suicide (FHoS), and the use of a violent suicidal means (VSM). Here, we added two groups of healthy first-degree biological relatives of suicide victims and depressed patients (*n* = 32). When comparing all suicide attempters and controls, very limited between-group differences were found in the two samples, and none in the meta-analysis. In contrast, a FHoS was associated with reduced volumes in bilateral temporal regions, right dorsolateral prefrontal cortex, and left putamen, several of these differences being observed across groups. VSM was associated with increased bilateral caudate (and left putamen) volumes. Some morphometric variations in cortico-subcortical networks may therefore be endophenotypes increasing the suicidal vulnerability, while others (notably in striatum) may modulate action selection. These results therefore confirm at the neural level two phenotypes at high lethal risk with a strong biological background, and uncover motives of heterogeneous findings in neuroimaging studies of suicidal behavior.

## Introduction

Suicide takes 800,000 lives each year in the world^1^. In spite of important efforts to improve our understanding of these complex acts, the identification of risk factors over the last 50 years have largely been limited to a few socio-demographic and clinical variables that lack predictive accuracy^2^. New research directions must be explored.

The last 20 years have witnessed the use of new techniques to clarify the pathophysiology of suicidal behavior. Among them, neuroimaging has been a tool of choice for the in vivo investigation of individuals with a past or recent history of suicide attempts or suicidal ideas^3^, and more recently relatives of suicide completers^4^. Since the first paper published in 2001, the present study found a total of 117 articles reporting neuroimaging findings in relation to suicidality. This growing literature opens the exciting perspective of the use of brain markers in risk prediction algorithms, and the development of brain marker-based therapeutic interventions to prevent suicide. However, neuroimaging studies have yielded too variable results to reach these goals to date. As for other mental disorders, this lack of consistency in results may partly stem from clinical and biological heterogeneities among studied populations. In the present article, we aimed at addressing this particular issue using structural brain imaging. To that purpose, we conducted a three-step investigation:

1. We conducted a “classical” comparison of T1 magnetic resonance imaging (MRI) sequences between patients with and without a history of suicidal acts, and healthy controls, in two independent samples.
2. In order to tackle the classical small sample size issue that may explain the lack of consistent results, we then run an exhaustive review of literature and used a meta-analytic approach. We only included significant foci extracted from corrected whole-brain analyses comparing patients with vs. without a history of suicidal behavior. We expected to identify the most robust patterns of structural brain alterations at the group level, notably in prefrontal cortex^5^ but not in subcortical regions^6,7^.
3. We explored the possibility that the lack of consistency in neuroimaging findings related to suicidal behavior may be explained by the variability in the biological mechanisms underlying different phenotypes. Here, we focused on the neural basis of two variables previously associated with biological/cognitive deficits and increasing the risk of suicide:

i. *A family history of suicide (FHoS)*. The heritability of suicidal behavior has been well established in family, twin and adoption studies, reaching 20% when comorbid disorders were taken into account^8^. The transmission of the suicidal risk appears to follow the transmission of particular traits such as impulsivity and aggression^8^. At the neurocognitive level, riskier choices and altered responses to risk during decision-making in ventral and dorsal prefrontal cortex have been found in both suicide attempters^9–11^ and first-degree biological relatives of suicide completers who themselves never attempted suicide^4,12^, suggesting that risky decision-making is an endophenotype of suicide. It is expected that some structural brain alterations be also inherited, notably in regions mentioned above. As not all suicide attempters have a FHoS, some brain alterations may only be found in suicide attempters with a FHoS, explaining some of the variability in neuroimaging studies. Following the endophenotype hypothesis, these FHoS-related alterations should furthermore be found across groups (suicide attempters, patient controls, and healthy controls).
ii. *A violent suicidal means (VSM)*. Use of a VSM increases the risk of future suicide death in comparison to non-violent suicidal methods (i.e., medication overdose and superficial wrist cutting)^13^. The choice of a VSM may be linked to particular biological mechanisms, including more serotonergic deficits^14^ (which are associated with increased risk of suicide death^15^), and riskier decision-making^9^ (which may be related to serotonergic deficits^16^). A history of VSM may therefore identify a particular biological phenotype among suicide attempters. Based on a previous study showing a link between the nucleus accumbens and suicidal lethality^7^, we hypothesized that this region may be particularly involved in the choice of a VSM in suicidal patients.

## Methods

### Participants

#### Suicide attempter samples

In both Montreal and Jena, three groups of male and female participants aged 18–57 years were recruited: (1) currently depressed patients with a personal history of attempted suicide (*Suicide attempters*; *n* = 17 in Montreal; *n* = 32 in Jena); (2) currently depressed patients with no personal history of suicide attempt (*Patient controls; n* = 26 in Montreal; *n* = 34 in Jena); and (3) non depressed controls with no personal or first-degree or second-degree family history of suicidal behavior (*Healthy controls; n* = 66 in Montreal; *n* = 34 in Jena). All suicide attempters and patient controls were depressed at the time of scanning, as determined by a Hamilton Depression Rating Scale (HDRS-21) score equal or higher than 20^17^, and a diagnosis of major depressive episode according to the Structured Clinical Interview for DSM-IV Axis I Disorders (SCID-I)^18^. In Montreal, outpatients were recruited from the Douglas Mental Health University Institute, while inpatients were recruited from the Department of Psychiatry and Psychotherapy at University Hospital Jena. Moreover, none of the participants was medicated at the time of the scanning in Montreal, while all patients were on stable antidepressant medication in Jena. In both samples, none of the healthy controls were taking any psychotropic medication. Suicide attempts were defined as any acts carried out with some intent to die and thus did not include non-suicidal self-injuries. A family history of suicidal behavior to the second biological degree was checked and recorded for all participants. In both samples, exclusion criteria comprised a lifetime history of schizophrenia or bipolar disorder, a history of alcohol/substance abuse or dependence spanning the previous 6 months, a major general medical condition requiring ongoing pharmacological treatment, a lifetime history of severe head trauma or central nervous system disorder, and contraindication to MRI. All participants were right-handed as confirmed by the Edinburgh Handedness Inventory^19^ in Montreal and the Annetts’ modified version of the Handedness Inventory^20^ in Jena. All participants were English-speaking or French-speaking natives in Montreal, and German-speaking natives in Jena. Informed written consent was obtained from all participants prior to their participation. Local ethics committees gave approval to these studies in both locations. Participants received 100 Canadian dollars or 10 euros per hour fees.

#### Suicide relative sample

In Montreal only, we recruited two groups of non-depressed relatives of patients: (1) 16 first-degree biological relatives of individuals who died from suicide in a context of major depressive disorder but not schizophrenia or bipolar disorder (*Suicide relatives*). Suicide relatives had no personal history of suicide attempt. (2) 17 first-degree biological relatives of depressed patients (*Patient relatives*) with no personal and family (to the second biological degree) history of suicidal acts. Patients suffered from major depressive disorder but not schizophrenia or bipolar disorder. All participants were aged 18–55, had to be normothymic at time of participation and free of psychotropic medication for the last 6 months. For more details, please check^4^.

### Image acquisition

#### Montreal

All scans were acquired at the Douglas Mental Health University Institute’s Cerebral Imaging Center (Montreal, Canada) using a Siemens Magnetom Trio (TIM System 3T) MRI scanner with a 12-channel head coil. High-resolution, whole-brain T1-weighted acquisition were collected using a magnetization prepared rapid gradient echo (MPRAGE) sequence with repetition time/echo time/flip angle = 2300/2.98 ms/9°, and a base resolution of 256 × 256, with 1 mm^3^ isotropic voxels resulting in acquisition time of 9.25 min.

#### Jena

The structural MR data were collected on a Siemens Magnetom Trio 3T whole body system equipped with a 64-element head matrix coil (TIM System 3T). High-resolution, whole-brain T1-weighted acquisition were collected using a MPRAGE sequence with repetition time/echo time/flip angle = 2300/3.03 ms/9°, and a base resolution of 256 × 256, with 1 mm³ isotropic voxels. All scans were inspected for motion artefacts and a neuroradiologist confirmed absence of gross pathological findings.

### Image analyses

An optimized voxel-based morphometry (VBM) approach^21^ was carried out using FSL-VBM^22^ with FSL^23^ toolbox, version 5.0.8. First, skull-striping procedures were implemented using Robust Brain Extraction (ROBEX) method^24^. Brain extracted images were automatically segmented in cerebrospinal fluid, and gray and white matter. A study-specific template was created registering gray matter images into Montreal Neurologic Institute (MNI) 152 space. In order to build such templates for pairwise group comparisons, a similar number of subjects within the largest group as in the smallest group were randomly chosen. Then, individual gray matter was registered to templates and modulated. Images were smoothed using full-width half-maximum of 3 mm. A General Linear Model was carried out with additional confounding variables as needed (e.g., age, location, group, and depression). Non-parametric tests were performed with 5000 permutations. False-positive outcomes were controlled by means of threshold free cluster enhancement. Survival clusters were reported for *p* < 0.05, corrected for multiple comparisons.

### Meta-analysis

#### Data sources

A review of literature until June 20th, 2017 was conducted on PubMed using the following entry terms: (magnetic resonance imaging OR diffusion magnetic resonance imaging OR magnetic resonance spectroscopy OR tomography, emission-computed OR positron-emission tomography OR tomography, emission-computed, single-photon) AND (suicide OR suicide, attempted OR suicidal ideation). This search yielded 599 results. An additional manual search through the references of extracted papers and existing reviews was conducted and twenty-two references were added.

#### Study screening and selection

Studies that met the following criteria were included: (1) published in English or French or German; (2) in a peer-reviewed journal; (3) using neuroimaging; (4) to investigate an association with suicidal behavior (as a primary or secondary objective); (5) patients were free from any known severe neurological condition or head trauma. Abstracts identified through the literature search were independently evaluated by two reviewers (GW and FJ) and selected by a consensus from all authors. Full articles were then obtained for final review. This selection yielded 117 articles (see full list in Supplementary Material).

**Fig. 1:**
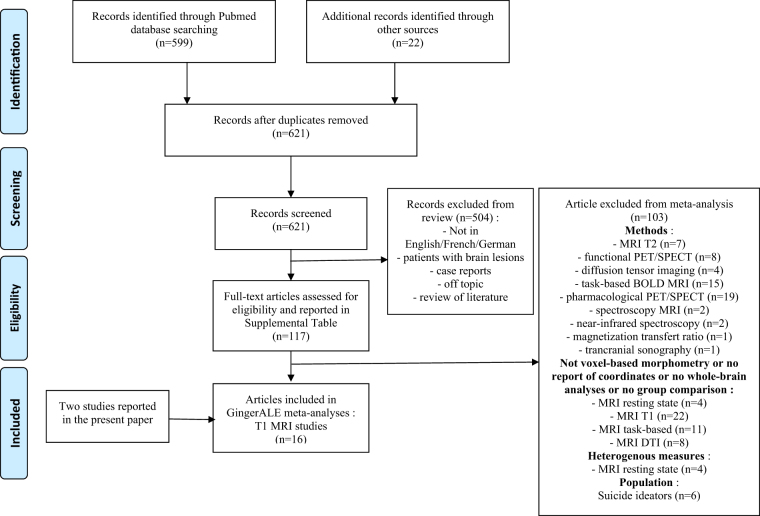
Flow chart for the meta-analysis

To run GingerALE meta-analyses, the additional following criteria were applied: (1) use of T1 MRI; (2) comparison of patients with vs. without a personal history of suicide attempt, or with vs. without a personal history of suicidal ideas; (3) whole-brain analyses with voxel coordinates reported. As studies investigating suicide ideators vs. non-ideators were rare (*n* = 6) and used various methods, we excluded these studies and focused on the comparison between suicide attempters and non-attempters. In total, 16 T1-structural (including Jena and Montreal samples) were retained for final analyses. See Supplementary Material, and Fig. 1.

### Data analyses

We used the activation likelihood estimation program GingerALE (version 2.3.6.)^25–28^. This program uses published peak voxel coordinates (when necessary, Talairach coordinates were transformed into MNI). Based on these coordinates, the program identifies commonly activated regions across subject groups. This analysis is driven by foci reported in the data sets and the manner in which they are grouped together. A Gaussian probability map of activation (called modeled activation maps) is computed using a mask, the foci reported for a given study and a Gaussian blur with a Full Width Half Maximum (FWHM) empirically derived from the size of the sample. The Gaussian maps are then used to generate a *p*-valued image. We first conducted analyses with an uncorrected *p* < 0.0001 to identify weak clusters. We then conducted analyses using a cluster-level inference *p* < 0.05 (with a voxel-level uncorrected level *p* < 0.001), and 1000 thresholding permutations. The ALE algorithm employed was the non-additive random effects model, and we chose the more conservative masking option. Sensitivity and secondary analyses were also performed as described below.

## Results

### Step 1: all suicide attempters vs. patient and healthy controls

See Table 1 for details about socio-demographic and clinical variables, and group comparisons.

**Table 1:** Comparison of socio-demographic and clinical variables between the groups for each of the three samples

	Montreal healthy controls		Montreal patient contro		Montreal suicide attempters		*F*/*χ*²	*P*	Post-hoc
	*n* = 66		*n* = 26		*n* = 17				
Gender, *n* males (%)	30	(45.5)	7	(28.0)	3	(17.6)	5.6	0.06	
Age, mean (SD)	32.3	(7.4)	41.0	(11.0)	37.6	(10.2)	9.7	<10^−3^	PC > HC
BDI score, mean (SD)	1.5	(2.3)	29.6	(11.5)	30.5	(11.5)	178.7	<10^−3^	PC, SA > HC
HDRS score, mean (SD)	1.7	(2.4)	28.4	(6.4)	28.5	(10.4)	282.7	<10^−3^	PC, SA > HC
Age at first depression (SD)	–	–	39.3	(11.8)	29.4	(9.9)	2.5	0.02	SA < PC
Number of depressive episodes (SD)	–	–	1.9	(1.2)	2.1	(1.1)	−0.3	0.7	
Family history of suicidal act, *n* (%)	–	–	7	(31.8)	3	(20.0)	0.6	0.3	
Number of suicidal act (SD)	–	–	–	–	1.4	(1.3)	–	–	
History of violent suicidal act, *n* (%)	–	–	–	–	2	(13.3)	–	–	
SIS score (SD)	–	–	–	–	18.4	(5.6)	–	–	
History of physical or sexual childhood trauma, *n* (%)	–	–	4	(19)	7	(50)	3.7	0.06	
	Jena healthy controls		Jena patient controls		Jena suicide attempters		*F*/*χ*²	*p*	Post-hoc
	*n* = 34		*n* = 34		*n* = 32				
Gender, *n* males (%)	9	(26.5)	9	(26.5)	9	(28.1)	0.03	1.0	
Age, mean (SD)	36.7	(9.8)	35.7	(11.9)	37.2	(11.8)	0.1	0.9	
BDI score, mean (SD)	2.0	(2.2)	29.0	(8.0)	26.7	(12.9)	98.7	<10^−3^	PC, SA > HC
HDRS score, mean (SD)	–	–	21.1	(9.6)	21.4	(9.9)	0.9	0.4	–
Age at first depression (SD)	–	–	29.6	(12.4)	29.6	(11.5)	0.01	1.0	
Number of depressive episodes (SD)	–	–	1.1	(1.2)	1.9	(2.2)	−1.8	0.08	
Family history of suicidal act, N (%)	–	–	5	(14.7)	6	(18.8)			
Number of suicidal act (SD)	–	–	–	–	1.2	(0.4)			
History of violent suicidal act, N (%)	–	–	–	–	11	(34.4)			
SIS score (SD)	–	–	–	–	20.3	(4.4)			
			Montreal patient relatives		Montreal suicide relatives		*F*/*χ*²	*p*	Post-hoc
			*n* = 17		*n* = 16				
Gender, *n* males (%)			7	(41.2)	8	(50.0)	0.3	0.9	
Age, mean (SD)			37.6	(8.5)	50.6	(9.2)	35.0	<10^−3^	SR > PR
BDI score, mean (SD)			1.6	(2.2)	1.9	(3.1)	0.2	0.8	
HDRS score, mean (SD)			1.7	(2.2)	2.3	(2.0)	0.4	0.7	

*BDI* Beck depression inventory, *HDRS* 21-item Hamilton rating scale for depression, *SIS* suicide intent scale, *SA* suicide attempters, *PC* patient controls, *HC* healthy controls, *PR* patient relatives, *SR* suicide relatives

In the Montreal sample, suicide attempters did not differ from patient and healthy controls in terms of gray and white matter volumes at the whole-brain corrected level. These results were not modified when co-variating with age and gender. In the Jena sample, suicide attempters showed reduced gray matter volume in comparison to patient controls in one very small cluster localized in the right dorsomedial prefrontal cortex (Brodmann Area [BA] 6; 6 voxels; MNI peak voxel: 2, 12, 50). Pooling samples (using location as a co-variable) did not yield any significant result.

### Step 2: suicide attempters vs. patient controls in the meta-analysis

Four studies (including our Montreal sample) showed no group difference between suicide attempters and non-attempter patients at a corrected whole-brain level of analysis^29–31^. The remaining 11 articles^5,32–41^ and our Jena sample represented 12 different studies, 693 subjects and 111 foci.

At uncorrected *p* < 0.0001 with all contrasts included, analyses revealed two clusters: one in the left insula (BA13), and one smaller in the left dorsomedial prefrontal cortex (BA6). None survived cluster correction. When analyses were restricted to the suicide attempters <non-attempters contrast (11 studies, 638 subjects, 105 foci), analyses at uncorrected *p* < 0.0001 revealed a similar picture with 4 significant clusters: two clusters in left anterior insula (BA13); and two small clusters in BA6. Again, none survived cluster correction. The opposite contrast was not analyzed due to the small number of studies (3 studies, 6 foci).

The above cluster-level results were unchanged when excluding the study in elderly^39^, or the three studies in psychosis^33,40,41^, or the study in borderline personality disorder^36^, or the two studies in adolescents and young adults^32,35^, or the study comparing high vs. low “suicidal risk” patients^38^, or our Jena sample. Finally, no significant difference was found between the four 3T-MRI vs. the eight 1.5/2T-MRI studies.

### Step 3: volumetric differences according to selected variables

#### Family history of suicide (FHoS)

**Fig. 2:**
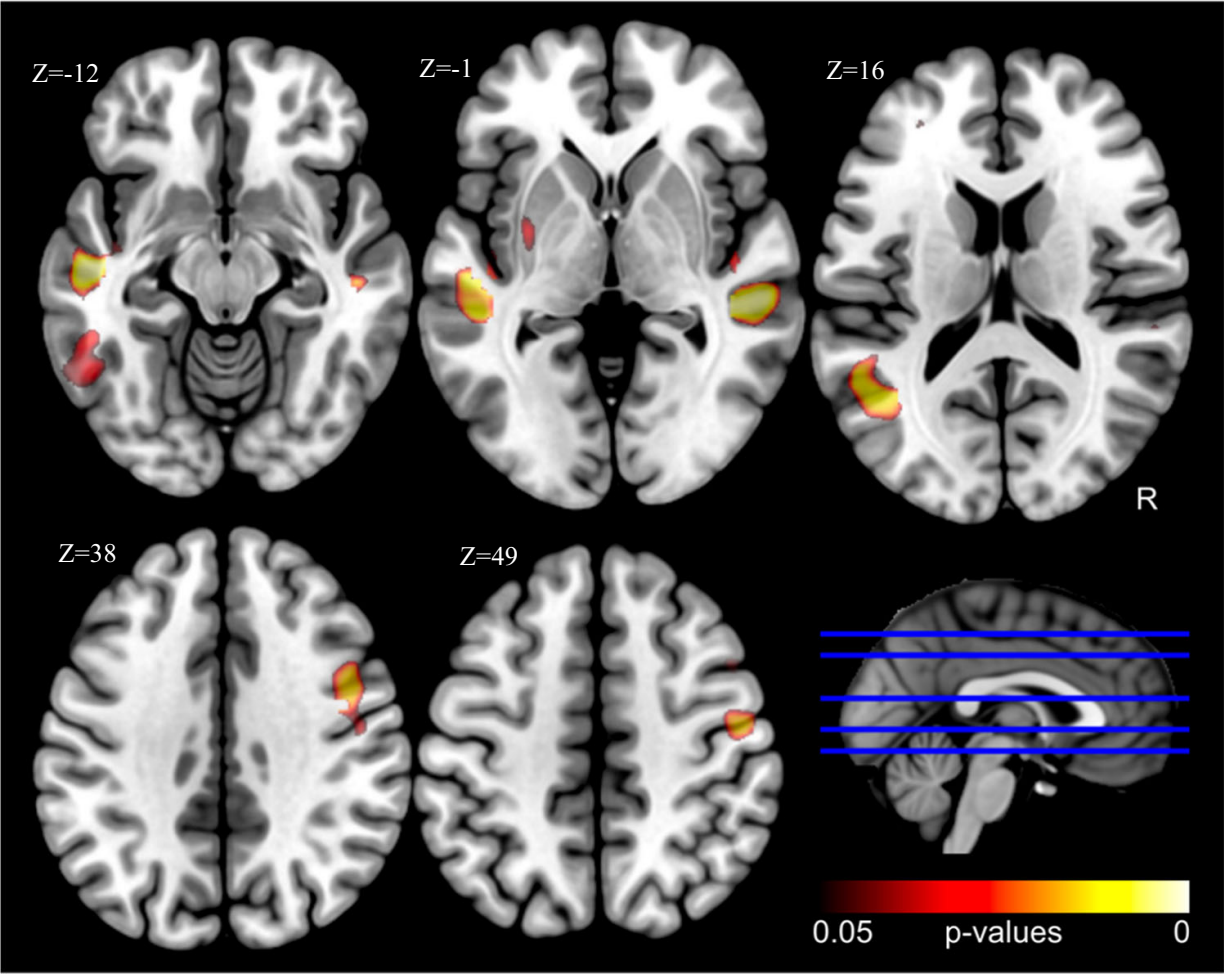
Reduced volumes in individuals with vs. without a family history of suicide (corrected *p* < 0.05; all groups combined, with site and depression as co-variables)

When suicide attempters, patient controls, and relatives were pooled (using both location and depression (yes/no) as co-variables), individuals with a FHoS at the first or second degree (*n* = 37 data available) in comparison to those without such history (*n* = 99) showed decreased gray matter volume in five clusters located in left (BA21/22; 1382 voxels; −50, −18, −16) and right (BA21/22; 732 voxels; 54, −32, −4) temporal gyri, left temporal gyrus extending toward fusiform gyrus (BA20/37; 238 voxels; −52, −44, −12), right dorsolateral prefrontal cortex (BA6/9; 472 voxels; 48, 2, 38), and left lentiform nucleus/putamen (97 voxels; −32, −6, 2) at corrected *p* < 0.05 levels (Fig. 2). Importantly for the endophenotype hypothesis, post-hoc analyses at uncorrected *p* < 0.001 level showed that several clusters, notably the left and right temporal clusters and the right dorsolateral prefrontal cluster, were found in all groups (healthy subjects, depressed patients, and suicide attempters) (see Supplementary Figure).

In patients without a FHoS, suicide attempters showed decreased signal in comparison to patient controls in left dorsomedial prefrontal cortex (BA6/8; 175 voxels; −4, 18, 58), while no group difference was found in those with a positive family history.

In order to explore the clinical correlates of the five clusters reported in the comparison between individuals with a positive and those with a negative FHoS, we extracted beta-values and computed the mean value for the whole cluster and the value for the peak voxel. HDRS scores were not correlated with any cluster in Montreal (*n* = 69) or Jena (*n* = 66). Similarly, Barratt Impulsiveness scale (BIS-11^42^) scores were not correlated with any cluster in Montreal (*n* = 34) or Jena (*n* = 65) or both combined. In Montreal where these data were available, the volume of the cluster located in the putamen was correlated with mental pain (worst during the last 15 days) measured by a visual analog scale^43^ (*n* = 37; mean: *r* = 0.35, *p* = 0.04; peak: *r* = 0.38, *p* = 0.02), and with a history of aggression during adolescence^44^ (*n* = 28; mean: *r* = 0.41, *p* = 0.03; peak: *r* = 0.46, *p* = 0.01).

#### Personal history of a violent suicidal means (VSM)

**Fig. 3:**
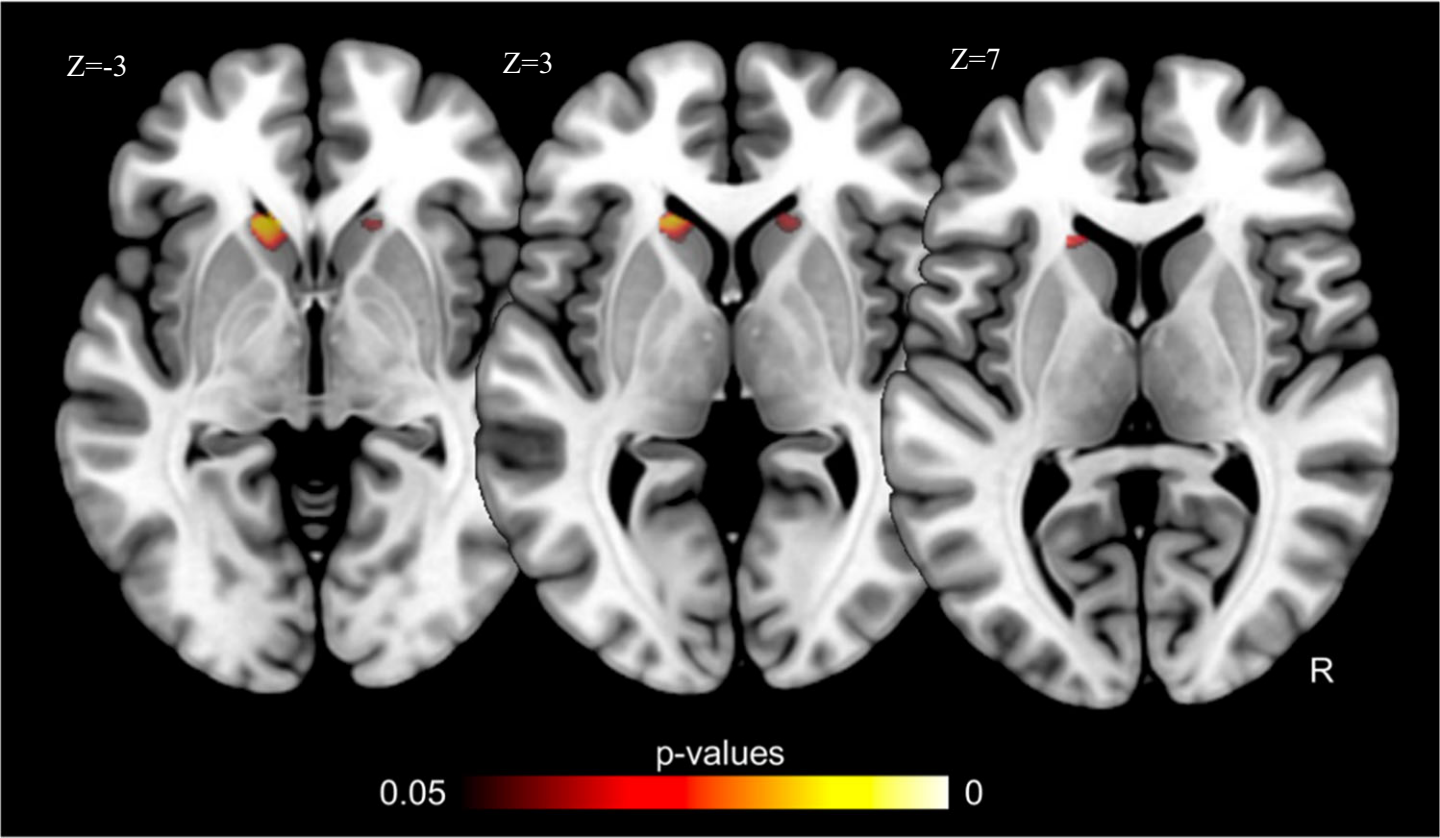
Increased volumes in patients who used a violent suicidal mean vs. those who used another suicidal means (corrected *p* < 0.05; using site as a co-variable)

After pooling Montreal and Jena samples (co-variating for site), suicide attempters with (*n* = 13) vs. without (*n* = 34) a history of VSM showed increased volume in left (25 voxels, −18, 24, −2) and right (21 voxels; 14, 22, −8) caudate nuclei at corrected *p* < 0.05 levels (Fig. 3). These clusters were larger (150 voxels; −12, 26, 0; and 45 voxels; 16, 24, 0; respectively) when the Jena sample was examined separately, possibly because this site provided the largest number of violent suicide attempts.

Caudate volumes were not correlated with HDRS, BIS-11, psychological pain or lifetime aggression scores.

In order to assess if these volumetric differences may stem from differences in suicidal intent, we run a similar analysis comparing attempters with high vs. low suicidal intent according to the median of the Suicidal Intent Scale^45^ for the most severe suicidal act (data available for 47 patients; median = 21). No significant group difference was found.

Finally, as FHoS and VSM tend to be associated^46^, we explored the possibility that some clusters identified in one comparison may be relevant to the other one. The two caudate clusters identified in the VSM analyses were not significant when comparing positive and negative FHoS. However, among the five clusters identified in the FHoS comparison, a logistic regression with site as a co-variable showed *increased* volume of the left putamen in suicide attempters who used a VSM vs. those who used a non-violent means (mean: *F* = 5.4, *p* = 0.02; peak: *F* = 6.4, *p* = 0.01).

## Discussion

Two main results emerged from the series of analyses presented here. First, we were unable to identify robust and consistent structural neuroimaging markers of suicidal behavior when all suicide attempters were analyzed together. While only patients with a sufficient level of intent when carrying out their suicidal act were recruited, group comparisons in two independent samples of depressed patients did not yield significant differences. Moreover, one meta-analysis of 12 different neuroimaging studies and almost 700 participants did not allow the identification of robust structural differences between attempters and non-attempters at cluster correction levels. Overall, these studies suggest a lack of morphometric differences when suicide attempters are considered as a group.

In contrast, two variables previously associated with cognitive or biological deficits and a higher risk of suicide death—FHoS and VSM—were significantly associated with volumetric alterations in specific brain regions. Several alterations related to a positive FHoS were furthermore observed in suicide attempters, patient controls, and healthy subjects, evoking endophenotypic features. These results suggest that these two variables may define two particular suicidal phenotypes with a specific biological and neurocognitive background. They may also explain a part of the variability in neuroimaging findings. Of note, an association between VSM and FHoS has been found in clinical studies^46^, suggesting a significant overlap between these two phenotypes. Neural mechanisms discussed below also support this overlap.

The heritability of suicidal behavior is well established. However, not all individuals who attempt or commit suicide have a FHoS^8^, and not all individuals with a FHoS will attempt suicide^47^. Individuals with a FHoS in comparison to those without such history have been shown to display a set of behavioral, cognitive, interpersonal, and brain impairments, including more risky decision-making^12^, altered responses of the prefrontal cortex to risk^4^, impaired inhibitory response to social stress^48^, and more impulsivity-aggression traits^47^. The present study suggests that a FHoS is additionally associated with structural brain alterations. We notably found reduced volumes of temporal and dorsolateral prefrontal cortices, and putamen. Interestingly, these regions previously showed moderate to high gray matter heritability in twin studies^49^. The role of these regions in the suicidal process will have to be clarified. To our knowledge, few neuroimaging studies linked these regions to specific functional impairments in suicide attempters. One study found reduced volumes of the temporal poles to be associated with lower empathy and increased risk of suicidal behavior in psychotic criminal offenders^33^. Another study reported a close cluster in right dorsolateral prefrontal cortex to be associated with risky decision-making in relatives of suicide victims^4^. These regions have all at different levels been implicated in processes relevant to suicidal behavior, notably social cognition and decision-making^50^, aggression^51^, or impulsivity and response inhibition^52^.

Importantly, secondary analyses showed *increased* putaminal volumes in violent suicide attempters. One hypothesis may be that decreased volumes in individuals with a FHoS who, for most of them never attempted suicide, may represent a protective factor. Reported morphometric alterations in this cortico-subcortical network in those with a FHoS may therefore represent neural conditions of both suicidal vulnerability and protection. It is important to note that significant differences emerged from the comparison between attempters and non-attempters in patients *without* a FHoS only, with reduced volumes of the dorsomedial prefrontal cortex in attempters supporting different neural mechanisms according to FHoS and the identification of different phenotypes within suicide attempters.

Individuals who use a VSM, relative to those who used non-violent methods, have a higher risk of ultimately dying from suicide^13^, even many years after their initial act. These individuals show more risky decision-making than controls^9^, and lower levels of the serotonin metabolite in the cerebrospinal fluid^14^. Our results additionally suggest increased volumes of the anterior part of the caudate in those with a history of VSM. Moreover, secondary analyses suggest increased putamen volumes as well. Few studies have reported consistent sub-cortical structural alterations in suicide attempters taken as a group. Wagner et al.^38^ found reduced right caudate volumes in high vs. low suicidal risk patients (risk comprises a personal or family history of suicidal behavior). Dombrovski et al.^53^ showed reduced putamen, but not caudate, gray matter in elderly suicide attempters vs. controls, a deficit correlated with higher delay discounting. Hwang et al.^39^ reported reduced lentiform nucleus volumes in elderly suicide attempters. Three studies, including very large ones, did not find any group differences^6,7,54^ and our meta-analysis did not find subcortical differences between suicide attempters and patient controls. Our findings suggest that structural alterations in the striatum may be associated with particular dimensions of the suicidal act—including the choice of a VSM—more than with suicidal behavior in general, explaining previous conflicting results in suicide attempters.

This dimensional approach makes sense in light of the literature. First, increased caudate volumes have previously been linked to violent offenses^55^ and more aggression^56^ in schizophrenia, and to antisocial choices in psychopaths^57^. On our side, we found significant positive correlations between putaminal volumes and both aggression and mental pain, two risk factors of suicide^58^. Serotonin may play a significant major role here and make the link between these various variables. Both nuclei are indeed modulated by serotonergic inputs^59^. Amphetamine abusers, who usually display chronic serotonergic deficits, showed increased striatal volumes^60^. Deficient serotonergic system is a known risk factor of aggression and violence in both humans and animals^44,61^, and of suicide^14^. Second, the region of the caudate nucleus reported in the present study is particularly involved in evaluating actions and in representing the expected outcome of actions, while the region of the putamen reported here is more involved in pain processing^62^. Impairment in ventral striatum, a region implicated in the encoding of the value of stimuli (in connection with the orbitofrontal and ventral prefrontal cortices)^62^, may also participate in the lethal decisional process, as suggested by a study showing negative correlation between the nucleus accumbens volumes and lethality of previous suicidal acts^7^. Again, the serotonergic system may be involved as tryptophan depletion leads to increased activity of the ventral striatum during the prediction of immediate reward^63^.

Dysfunctional dorsal and ventral striatum, through reciprocal connections with the cortex and under deficient serotonergic inputs, may therefore participate in several steps of the suicidal process. Ultimately, deficient activity of the striatum may also be predictive of future suicide as shown by the only longitudinal neuroimaging study^64^. One general hypothesis may be that excessive value given to the current stressful situation experienced by the individual^65^ (in relation to ventral striatum and orbitofrontal cortex^66,67^) in combination with deficient regulation of pain^58^ (in relation to the posterior putamen^68^ and possibly the posterior insula^69^ and dorsomedial prefrontal cortex^70^), and poor assessment of outcomes of actions (in relation to caudate, and dorsolateral^4,67^ and ventromedial prefrontal cortices^4,71^) may lead to mental pain, the search for immediate reward (sedation of pain), reduced inhibition and a tendency for aggressive and violent responses. The choice of suicide among potential responses may depend on the more evolutionary-developed prefrontal cortex. This hypothesis is in agreement with Dombrovski and Hallquist^72^ who recently proposed a predominance of pavlovian processes (stimulus-response association) over goal-orientated processes (taking into account outcomes).

The strengths of the present study comprise the analysis of two independent samples, an exhaustive review of literature, the conduct of one meta-analysis, and the possibility to test the neural basis of two important phenotypes. Weaknesses include small sample sizes, which may have explained some negative results; the exclusion of several structural neuroimaging studies from analyses (e.g., those based on surface-based morphometry, or region-of-interest analyses); and the impossibility to run analyses in suicidal ideators (with no history of suicidal act) in order to disentangle the mechanisms of suicidal ideas vs. acts. Moreover, pooling samples with non-balanced groups, which is the case here, and covariating for location reduce the variance induced by the use of different scanners but also limit the identification of group differences^73^.

In conclusion, this study suggests that considering suicidal behavior as a whole is unlikely to provide insight into the biological substratum and identify biomarkers of suicidal risk. In contrast, a FHoS and the choice of a VSM may be associated with what could be considered “neural phenotypes”. More variables that are associated with brain structural variations and have a significant influence on the risk of suicidal acts (e.g., childhood abuse) will have to be studied. Ultimately, this will allow a better characterization of the mechanisms in play in suicidal behavior at the network level and, hopefully, more personalized care and better prevention.

## Electronic supplementary material

Supplemental Material
