# Supplementary material for: Neuroimaging-informed phenotypes of suicidal behavior: a family history of suicide and the use of a violent suicidal means
Source: Transl Psychiatry. 2018 Jun 19;8:120. doi: 10.1038/s41398-018-0170-2 (PMC6008434; doi:10.1038/s41398-018-0170-2)

**SUPPLEMENTAL MATERIAL**

**Table. Review of literature of neuroimaging studies on suicidal behavior.**

| **Article** | **Methods 1** | **Methods 2** | **Main comorbid disorder** | **Age group** | **Gender** | **Main suicidality contrast** | **Note** | **Motive of non-inclusion in meta-analysis** |
| --- | --- | --- | --- | --- | --- | --- | --- | --- |
| **T1-structural MRI** |  |  |  |  |  |  |  |  |
| (Gifuni et al., 2017) | MRI 1.5T | Freesurfer (volume), corpus callosum | MDD and BD (euthymic) | ADULTS | M/F | SA vs. PC vs. HC | 2 independent samples pooled | Freesurfer, ROI |
| (Duarte et al., 2017) | MRI 1.5T | SPM8 | BD I (euthymic) | ADULTS | M/F | SA vs. PC vs. HC | No whole brain group difference; only for ROI and high lethality attempters |  |
| (Johnston et al., 2017) | MRI 3T | SPM5 | BD (various states) | ADOLESCENTS/YOUNG ADULTS | M/F | SA vs. PC |  |  |
| (Harenski et al., 2017) | MRI 1.5T | SPM12 | Criminal offenders with psychotic symptoms | ADULTS | M | SA vs. 3 control groups |  |  |
| (Besteher et al., 2016) | MRI 1.5T | Freesurfer (thickness and folding) | SCZ | ADULT | M/F | SA vs. PC |  | Freesurfer |
| (Fradkin et al., 2016) | MRI 3T | Freesurfer (thickness), ROI and WB | MDD (various levels of depression) | ADOLESCENTS | M/F | SA vs. HC |  | Freesurfer, no PC |
| (Gosnell et al., 2016) | MRI 3T | Freesurfer (volume), various ROI | Mostly MDD, also BD and others; mild to severe depression | ADULTS | M/F | SA vs. PC vs. HC | Recent SA | Freesurfer, ROI |
| (Lee et al., 2016b) | MRI 1.5T | SPM8 | MDD (depressed) | ADULTS | M/F | SA vs. PC |  |  |
| (Gifuni et al., 2016) | MRI 1.5T | Freesurfer (volume), subcortical nuclei | MDD and BD (euthymic) | ADULTS | M/F | SA vs. PC vs. HC | 3 independent samples pooled | Freesurfer, ROI |
| (Ding et al., 2015) | MRI 1.5T | SPM8 & Freesurfer (volume), prefrontal cortex | MDD and BD (euthymic) | ADULTS | M/F | SA vs. PC vs. HC | 3 independent samples pooled, SPM Whole brain analyses reported |  |
| (Kim et al., 2015) | MRI 3T | SPM5 | Panic disorder | ADULT | M/F | SA vs. PC | No whole brain group difference |  |
| (Colle et al., 2015) | MRI 1.5&3T | SACHA, hippocampus | MDD (depressed) | ADULTS | M/F | SA vs. PC |  | ROI |
| (Pan et al., 2015) | MRI 3T | Freesurfer (thickness & volume) | MDD (depressed) | ADOLESCENTS | M/F | SA vs. PC |  | Freesurfer |
| (Benedetti et al., 2014) | MRI 3T | SPM8 | BD I | ADULTS | M/F | Interaction early stress, 5HLTTLPR and volume |  | No group comparison reported |
| (Lijffijt et al., 2014) | MRI 1.5&3T | Freesurfer (volume), ROI | BD (various states) | ADULTS | F | SA vs. PC | Effect of hospitalization was found | Freesurfer, ROI |
| (Peng et al., 2014) | MRI 3T | SPM8 | MDD (depressed) | YOUNG ADULTS | M/F | SA vs. PC vs. HC |  |  |
| (Soloff et al., 2014a) | MRI 1.5T | SPM8 | BPD | ADULTS | M/F | all SA, high vs. low lethality |  | No PC |
| (Giakoumatos et al., 2013) | MRI 3T | Freesurfer (volume) | SCZ, SZA, BD with psychotic features | ADULTS | M/F | SA vs. PC vs. HC | Also high vs. low lethality | Freesurfer |
| (Wagner et al., 2012) | MRI 1.5T | Freesurfer (thickness) | MDD (depressed) | ADULTS | M/F | High vs. low suicide risk vs. HC | Risk defined as SA or family history of suicide | Freesurfer |
| (Nery-Fernandes et al., 2012) | MRI 1.5T | ANALYZE AVW 7.0, corpus callosum | BD I (euthymic) | ADULTS | M/F | SA vs. PC vs. HC |  | ROI |
| (Soloff et al., 2012) | MRI 1.5T | SPM5, 9 ROIs | BPD | ADULTS | M/F | SA vs. PC vs. HC | Also high vs. low lethality, Included as many ROIs assessed |  |
| (Dombrovski et al., 2012) | MRI 3T | ALP, basal ganglia | MDD (depressed) | ELDERLY | M/F | SA vs. PC vs. HC | Also correlation with delay discounting | ROI |
| (Baldacara et al., 2011) | MRI 1.5T | SPM5, cerebellum | BD I (euthymic) | ADULTS | M/F | SA vs. PC | Secondary analyses | ROI |
| (Benedetti et al., 2011) | MRI 3T | SPM5 | BD (depressed) | ADULTS | M/F | SA vs. PC | Also effect of lithium |  |
| (Cyprien et al., 2011) | MRI 1.5T | Manually traced, corpus callosum | Mostly MDD, also anxiety and BD | ELDERLY | M/F | SA vs. PC vs. HC | Community cohort | ROI |
| (Goodman et al., 2011) | MRI 3T | Anterior cingulate cortex | BPD & MDD | ADOLESCENTS | M/F | Correlation number suicidal act | Secondary analyses | ROI, No group comparison |
| (Spoletini et al., 2011) | MRI 3T | FSL 4.1, subcortical nuclei | SCZ | ADULTS | M/F | SA vs. PC vs. HC |  | ROI |
| (Wagner et al., 2011) | MRI 1.5T | SPM2 | MDD (depressed) | ADULTS | M/F | High vs. low suicide risk vs. HC | Risk defined as SA or family history of suicide |  |
| (Vang et al., 2010) | MRI 1.5T | Freesurfer (volume), subcortical nuclei | Various affective disorders | ADULTS | M/F | SA vs. HC |  | Freesurfer, ROI, no PC |
| (Hwang et al., 2010) | MRI 2T | SPM2 | Late-onset depression | ELDERLY | M | SA vs. PC vs. HC |  |  |
| (Matsuo et al., 2010) | MRI 1.5T | Manually traced, corpus callosum | BD | ADULTS | F | SA vs. PC vs. HC |  | ROI |
| (Jia et al., 2010) | MRI 3T | SPM2 | MDD (depressed) | ADULTS | M/F | SA vs. PC vs. HC | No whole brain group difference |  |
| (Rusch et al., 2008) | MRI 1.5T | SPM2 | SCZ | ADULTS | M/F | SA vs. PC vs. HC |  |  |
| (Aguilar et al., 2008) | MRI 1.5T | SPM5 | SCZ | ADULTS | M | SA vs. PC |  |  |
| (Jovev et al., 2008) | MRI 1.5T | Manually traced, pituitary gland | BPD | ADOLESCENTS | M/F | Correlation parasuicidal act |  | ROI, no group comparison |
| (Monkul et al., 2007) | MRI 1.5T | BRAINS, 4 ROIs | MDD (various levels of depression) | ADULTS | F | SA |  | ROI |
| **Task-based BOLD functional MRI** |  |  |  |  |  |  |  |  |
| (Kim et al., 2017) | MRI 3T | SPM5, emotional faces and suicidal means | Mostly MDD | ADULTS | M/F | SA vs. HC | Interaction with methylation of CACNA1C gene |  |
| (Olié et al., 2017) | MRI 1.5T | SPM12, Cyberball Game | MDD and BD (euthymic) | ADULTS | F | SA vs. PC vs. HC |  |  |
| (Johnston et al., 2017) | MRI 3T | SPM5/8, connectivity from amygdala during emotional faces | BD (various states) | ADOLESCENTS/YOUNG ADULTS | M/F | SA vs. PC |  |  |
| (Quevedo et al., 2016) | MRI 3T | SPM8, self-face recognition | Depression | ADOLESCENTS | M/F | high vs. low suicidality (SI+SA) vs. HC |  |  |
| (Minzenberg et al., 2016) | MRI 1.5T | SPM8/AFNI, Continuous Performance Task, Anterior cingulate cortex connectivity | MDD & BD with psychotic features | YOUNG ADULTS | M/F | SI vs. non SI, SA vs. PC |  |  |
| (Ding et al., 2016) | MRI 3T | AFNI/FSL, Iowa Gambling Task | MDD in family | ADULTS | M/F | Suicide Relatives |  |  |
| (Richard-Devantoy et al., 2016a) | MRI 3T | SPM12, Go/NoGo | MDD (depressed) | ADULTS | M/F | SA vs. PC vs. HC | No whole brain group difference |  |
| (Minzenberg et al., 2015b) | MRI 1.5T | SPM8/AFNI, Stroop task | SCZ (recent-onset) | YOUNG ADULTS | M/F | SI vs. non SI, SA vs. PC |  |  |
| (Olié et al., 2015) | MRI 1.5T | SPM8, Iowa Gambling Task & emotional faces, ROI | MDD and BD (euthymic) | ADULTS | M | SA vs. PC vs. HC |  |  |
| (Vanyukov et al., 2016) | MRI 3T | AFNI/FSL, Delay discounting task | MDD (depressed) | ELDERLY (AND YOUNGER ADULTS) | M/F | SA vs. PC vs. HC |  |  |
| (Minzenberg et al., 2015c) | MRI 1.5T | SPM8/AFNI, Continuous Performance Task | MDD & BD with psychotic features | YOUNG ADULTS | M/F | SI vs. non SI, SA vs. PC |  |  |
| (Minzenberg et al., 2015a) | MRI 1.5T | SPM8/AFNI, Continuous Performance Task, Anterior cingulate cortex connectivity | SCZ (recent-onset) | YOUNG ADULTS | M/F | SI vs. non SI, SA vs. PC |  |  |
| (Vanyukov et al., 2015) | MRI | AFNI/FSL, emotional faces | MDD (depressed) | ELDERLY | M/F | SA vs. PC vs. HC | No whole brain group difference |  |
| (Minzenberg et al., 2014) | MRI 1.5T | SPM8/AFNI, Continuous Performance Task | SCZ (recent-onset) | YOUNG ADULTS | M/F | SI vs. non SI, SA vs. PC |  |  |
| (Marchand et al., 2013b) | MRI 3T | SPM5, motor activation task | MDD (depressed) | ADULTS | M | Correlation with self-harm |  |  |
| (Dombrovski et al., 2013) | MRI 3T | AFNI, probabilistic reversal learning task | MDD (depressed) | ELDERLY | M/F | SA vs. (PC+HC) | Coordinates provided by author |  |
| (Zhang et al., 2013) | MRI 3T | SPM8, N-Back task | SCZ (first episode) | YOUNG | M/F | high vs. low risk vs. HC |  |  |
| (Marchand et al., 2013a) | MRI 3T | SPM5, motor activation task | MDD & BD II (depressed) | ADULTS | M/F | Correlation curent SI | Secondary analyses |  |
| (Pan et al., 2013b) | MRI 3T | SPM8/FSL, Emotional faces and connectivity (PPI) | MDD (depressed) | ADOLESCENTS | M/F | SA vs. PC vs. HC |  |  |
| (Pan et al., 2013a) | MRI 3T | SPM5, Iowa Gambling Task | MDD (depressed) | ADOLESCENTS | M/F | SA vs. PC vs. HC |  |  |
| (Marchand et al., 2012) | MRI 3T | SPM5, motor activation task functional conectivity | MDD (depressed) | ADULTS | M | Correlation with self-harm |  |  |
| (Marchand et al., 2011) | MRI 3T | SPM, motor activation task functional conectivity | BDII (depressed) | ADULTS | M | Correlation SI |  |  |
| (Pan et al., 2011) | MRI 3T | SPM5, Go/NoGo | MDD (depressed) | ADOLESCENTS | M/F | SA vs. PC vs. HC |  |  |
| (Reisch et al., 2010) | MRI 1.5T | Brain Voyager, Recall of suicidal episode | Depression | ADULTS | F | All recent SA, within group analyses |  |  |
| (Jollant et al., 2010) | MRI 1.5T | SPM5, Iowa Gambling Task | MDD (euthymic) | ADULTS | M | SA vs. PC vs. HC | No whole brain group difference |  |
| (Jollant et al., 2008) | MRI 1.5T | XBAM, emotional faces | MDD (euthymic) | ADULTS | M | SA vs. PC vs. HC |  |  |
| **Diffusion Tensor Imaging (DTI)** |  |  |  |  |  |  |  |  |
| (Lischke et al., 2017) | MRI 3T | FSL, corpus callosum | BPD | ADULTS | F | SA vs. PC vs. HC | Secondary analyses |  |
| (Chase et al., 2017) | MRI 3T | FSL, cingulum bundle | mixed | YOUNG ADULTS | M/F | SI vs. HC |  |  |
| (Johnston et al., 2017) | MRI 3T | SPM5, whole brain | BD (various states) | ADOLESCENTS/YOUNG ADULTS | M/F | SA vs. PC |  |  |
| (Cyprien et al., 2016) | MRI 1.5T | FSL, corpus callosum | MDD and BD (euthymic) | ADULTS | F | Complex analyses according to BD and MDD |  |  |
| (Myung et al., 2016) | MRI 3T | FSL/Freesurfer, whole brain graph theory with Network Based Statistics (NBS) | MDD (depressed) | ADULTS | M/F | SI vs. non SI vs. HC |  |  |
| (Lee et al., 2016a) | MRI 3T | FSL, whole brain | SCZ | ADULTS | M/F | SA vs. PC |  |  |
| (Bijttebier et al., 2015) | MRI 3T | ExploreDTI, whole brain graph theory with Network Based Statistics (NBS) | MDD (euthymic) | ADULTS | M/F | SA vs. PC vs. HC |  |  |
| (Kim et al., 2015) | MRI 3T | FSL, whole brain | Panic disorder | ADULTS | M/F | SA vs. PC |  |  |
| (Olvet et al., 2014) | MRI 3T | FSL/Freesurfer, ROI anterior cingulate and some prefrontal regions | MDD (depressed) | ADULTS | M/F | SA vs. PC vs. HC |  |  |
| (Jia et al., 2014) | MRI 3T | Trackvis, anterior limb of the internal capsule | MDD (depressed) | ADULTS | M/F | SA vs. PC vs. HC |  |  |
| (Mahon et al., 2012) | MRI 1.5T | FSL, whole brain | BD I (mainly euthymic) | ADULTS | M/F | SA vs. PC vs. HC |  |  |
| (Jia et al., 2010) | MRI 3T | SPM2, whole brain | MDD (depressed) | ADULTS | M/F | SA vs. PC vs. HC |  |  |
| **Resting State MRI** |  |  |  |  |  |  |  |  |
| (Kang et al., 2017) | MRI 1.5T | SPM8/AFNI, functional connectivity with amygdala | MDD (depressed) | ADULTS | M/F | SA vs. PC |  |  |
| (Du et al., 2017) | MRI 3T | SPM8, RS functional connectivity with rostral anterior cingulate cortex | MDD (depressed) | ADULTS | M/F | SI vs. non SI vs. HC |  |  |
| (Chase et al., 2017) | MRI 3T | SPM8, functional connectivity with anterior and posterior cingulate | mixed | YOUNG ADULTS | M/F | SI vs. HC | Secondary analyses in SA vs. PC |  |
| (Zhang et al., 2016) | MRI 3T | DPARSFA, default-mode network | Depression | ADOLESCENTS/YOUNG ADULTS | M/F | SA vs. PC vs. HC |  |  |
| (Cao et al., 2016) | MRI 3T | REST,fractional amplitude of low-frequency fluctuation (fALFF) | Depression | ADOLESCENTS/YOUNG ADULTS | M/F | SA vs. PC vs. HC |  |  |
| (Cao et al., 2015) | MRI 3T | REST/SPM5/DPARSFA, regional homogeneity (Rho) | No diagnosis | ADOLESCENTS/YOUNG ADULTS | M/F | SA vs. HC |  |  |
| (Cullen et al., 2014) | MRI 3T | FSL/Freesurfer, functional connectivity with amygdala | MDD (depressed) | ADOLESCENTS | M/F | correlation with suicidality | Secondary analyses |  |
| (Fan et al., 2013) | MRI 3T | REST,fractional amplitude of low-frequency fluctuation (fALFF) | MDD (depressed) | ADULTS | M/F | SA vs. PC vs. HC |  |  |
| **T2-structural MRI** |  |  |  |  |  |  |  |  |
| (Sachs-Ericsson et al., 2014) | MRI 1.5T | MrX, white matter esions | MDD (depressed) | ELDERLY | M/F | SA vs. PC | follow-up study |  |
| (Pompili et al., 2008) | MRI 1.5T | Two blind raters, white matter hyperintensities | MDD and BD (depressed) | ADULTS | M/F | SA vs. PC |  |  |
| (Pompili et al., 2007) | MRI 1.5T | Two blind raters, white matter hyperintensities | MDD and BD (depressed) | ADULTS | M/F | SA vs. PC |  |  |
| (Ehrlich et al., 2005) | MRI ?T | One blind rater, white matter hyperintensities | MDD | YOUNG ADULTS | M/F | SA vs. PC |  |  |
| (Ehrlich et al., 2004) | MRI 1.5T | One blind rater, white matter hyperintensities | MDD, BD, Psychosis, various | CHILDREN  /ADOLESCENTS | M/F | SA vs. PC |  |  |
| (Ehrlich et al., 2003) | MRI ?T | Unknown method, white matter hyperintensities | MDD among others not indicated | CHILDREN  /ADOLESCENTS | M/F | SA vs. PC |  |  |
| (Ahearn et al., 2001) | MRI 1.5T | Two blind raters, white matter hyperintensities | MDD (depressed) | ADULTS  /ELDERLY | M/F | SA vs. PC |  |  |
| **Functional PET/SPECT** |  |  |  |  |  |  |  |  |
| (van Heeringen et al., 2017) | PET [18F]-fluoro-2-deoxyglucose (FDG) | SPM12, resting state | MDD (depressed) | ADULTS | M/F | SI with plan vs. SI no plan vs. PC vs. HC |  |  |
| (Ballard et al., 2014) | PET [18F]-fluoro-2-deoxyglucose (FDG) | SPM5, ROI, baseline and after ketamine | Treatment resistant MDD | ADULTS | M/F | Correlation suicide ideation |  |  |
| (Sublette et al., 2013) | PET [18F]-fluoro-2-deoxyglucose (FDG) | SPM5, after placebo or fenfluramine | MDD and BD (depressed) | ADULTS | M/F | SA vs. PC |  |  |
| (Willeumier et al., 2011) | SPECT technetium-99m exametazime | SPM8, Conner’s Continuous Performance Test | various, mostly depressed | ADULTS | M/F | Future suicide completion | Follow up study |  |
| (Fountoulakis et al., 2004) | SPECT 99mTc-hexamethylpropyleneamine oxime (HMPAO) | Unknown, ROI, resting state | MDD (depressed) | ADULTS | M/F | PC, SI vs. non SI |  |  |
| (Amen et al., 2009) | SPECT technetium-99m exametazime | SPM2, Conner’s Continuous Performance Test | various, mostly depressed | ADULTS | M/F | Future suicide completion | Follow up study |  |
| (Oquendo et al., 2003) | PET | SPM96, ROI, after placebo or fenfluramine | MDD (depressed) | ADULTS | M/F | All SA, high vs. low lethality |  |  |
| (Audenaert et al., 2002) | SPECT 99mTc-Ethyl Cystine Dimer | SPM99, verbal fluency | MDD (depressed) | ADULTS | M/F | SA vs. HC |  |  |
| **Pharmacological PET/SPECT** |  |  |  |  |  |  |  |  |
| (Oquendo et al., 2016) | FLIRT, PET [11C]WAY- 100635, [11C]DASB (5HT1A & 5HTT) | Midbrain | MDD (depressed) | ADULTS | M/F | Future SA, future SI | Follow up study over 2 years |  |
| (Miller et al., 2016) | FLIRT, PET [11C]DASB (5HTT) | 6 ROIs | BD (depressed) | ADULTS | M/F | SA vs. PC | Secondary analyses |  |
| (Yeh et al., 2015) | PET 4-[18F]-ADAM (5HTT) | 4 ROIs | MDD (depressed) | ADULTS | M | Correlation with SI | Male conscripts, secondary analyses |  |
| (Kolla et al., 2016) | PET [11C] harmine (MAOA) | Prefrontal cortex and anterior cingulate | BPD, MDD | ADULTS | F | Correlation with sucidality | Secondary analyses |  |
| (Sullivan et al., 2015) | PET [11C]WAY-100635 (5HT1A) | 4 prefrontal ROIs + midbrain | MDD (depressed) | ADULTS | M/F | SA vs. PC |  |  |
| (Yeh et al., 2014) | PET 4-[18F]-ADAM (5HTT) | 4 ROIs | MDD (depressed) | ADULTS | M/F | Recent SA vs. PC | Within 2 weeks of SA |  |
| (Soloff et al., 2014b) | PET [(18)F]altanserin (5HT2A) | 9 ROIs | BPD | ADULTS | M/F | SA vs. PC | Secondary analyses |  |
| (Nye et al., 2013) | PET [11 C]-ZIENT (5HTT) | 8 ROIs | MDD (depressed) | ADULTS | M/F | SA vs. HC |  |  |
| (Miller et al., 2013) | PET [11C]DASB (5HTT) | 6 ROIs | MDD (depressed) | ADULTS | M/F | SA vs. PC |  |  |
| (Soloff et al., 2007) | PET [18F] altanserin (5HT2A) | 4 ROIs | BPD | ADULTS | F | SA vs. HC | Secondary analyses |  |
| (Cannon et al., 2006) | PET [11C]DASB (5HTT) | 8 ROIs | BD (depressed) | ADULTS | M/F | SA vs. PC | Secondary analyses |  |
| (Oquendo et al., 2007) | PET [11C]( )-McNeil 5652 (5HTT) | 6 ROIs | BD (depressed) | ADULTS | M/F | SA vs. PC | Secondary analyses |  |
| (Ryding et al., 2006) | SPECT 123I-β-CIT (5HTT and DAT) | ROIs | Mixed | ADULTS | M/F | SA vs. HC |  |  |
| (Parsey et al., 2006) | PET [11C]McN 5652 (5HTT) | 6 ROIs | MDD (depressed) | ADULTS | M/F | SA vs. PC vs. HC | Secondary analyses |  |
| (Leyton et al., 2006) | PET a-[11C]methyl-L-tryptophan | 3 ROIs | Mixed | ADULTS | M/F | SA vs. HC |  |  |
| (Lindstrom et al., 2004) | SPECT 123I-β-CIT (5HTT and DAT) | ROIs | Mixed | ADULTS | M/F | SA vs. HC |  |  |
| (Meyer et al., 2003) | PET [18F]Setoperone (5HT2) | ROIs, before and after fenfluramine or clonidine | MDD (depressed) | ADULTS | M/F | SA with chronic SI vs. HC | Secondary analyses |  |
| (van Heeringen et al., 2003) | SPECT [123I]5-I-R9115 (5HT2A) | 12 ROIs | Mixed | ADULTS | M/F | SA vs. HC |  |  |
| (Audenaert et al., 2001) | SPECT [123I]5-I-R9115 (5HT2A) | 12 ROIs | Mixed | ADULTS | M/F | SA vs. HC |  |  |
| **Other methods** |  |  |  |  |  |  |  |  |
| (Jollant et al., 2017) | MRI, Proton Magnetic Resonance Spectroscopy | Right dorsolateral prefrontal cortex | MDD (depressed) | ADULTS | M/F | SA vs. PC vs. HC | And correlation suicidal ideation and mental pain |  |
| (Rocha et al., 2015) | MRI, Proton Magnetic Resonance Spectroscopy | Medial orbitofrontal cortex | BD I (euthymic) | ADULTS | M/F | SA vs. PC vs. HC |  |  |
| (Tsujii et al., 2017) | 52-channel Near infra-red spectroscopy | Mainly prefrontal, verbal fluency task | MDD (depressed) | ADULTS | M/F | SA vs. PC vs. HC |  |  |
| (Pu et al., 2015) | 52-channel Near infra-red spectroscopy | Prefrontal, verbal fluency task | MDD (depressed) | ADULTS | M/F | SI vs. non SI |  |  |
| (Chen et al., 2015) | MRI, Magnetization transfer imaging | SPM8, resting state | MDD (depressed) | ADULTS | M/F | SA vs. PC vs. HC |  |  |
| (Budisic et al., 2010) | Transcranial sonography | Two blinded physicians | MDD (depressed) | ADULTS | ? | SI vs. non SI |  |  |

*Footnotes*: MDD : Major Depressive Disorder; BD : Bipolar Disorder; BPD : Borderline Personality Disorder; SCZ : Schizophrenia; SZA : Schizoaffectif Disorder; SA : Suicide attempters; SI : Suicide Ideators; PC : Patient Controls; HC : Healthy Controls; M : Male; F : Female; ROI : Region-of-Interest; MRI : Magnetic Resonance Imaging; SPECT : Single Photon Emission Computed Tomography; PET : Positron Emission Tomography; 5HT_2A_: Serotonin 2A receptor; 5HTT : Serotonin Transporter; MAOA : Monoamine Oxydase-A; WAY: ^3^H-(*N*-(2-(1-(4-(2-methoxyphenyl)-1-piperazinyl)ethyl)-*N*-(2-pyridyl) cyclohexane-carboxamide); DASB: 3-amino-4-(2-dimethylaminomethylphenylsulfanyl)-benzonitrile; SPM: Statistical Parametric Mapping; AFNI: Analyses of Functional NeuroImages; FSL: FRMIB Software Library.

**Existing reviews of literature that were consulted for the review**

(Richard-Devantoy et al., 2016b; Zhang et al., 2014; Martin et al., 2015; Sobanski et al., 2015; van Heeringen et al., 2014; Cox Lippard et al., 2014; Desmyter et al., 2011; Jollant et al., 2011; Grangeon et al., 2010).

**References from the review of literature and meta-analysis**

Aguilar, EJ, Garcia-Marti, G, Marti-Bonmati, L, Lull, JJ, Moratal, D, Escarti, MJ, Robles, M, Gonzalez, JC, Guillamon, MI, Sanjuan, J, 2008, “Left orbitofrontal and superior temporal gyrus structural changes associated to suicidal behavior in patients with schizophrenia” *Prog Neuropsychopharmacol Biol Psychiatry* **32**(7) 1673–1676

Ahearn, EP, Jamison, KR, Steffens, DC, Cassidy, F, Provenzale, JM, Lehman, A, Weisler, RH, Carroll, BJ, Krishnan, KR, 2001, “MRI correlates of suicide attempt history in unipolar depression.” *Biol Psychiatry* **50**(4) 266–270

Amen, DG, Prunella, JR, Fallon, JH, Amen, B, Hanks, C, 2009, “A comparative analysis of completed suicide using high resolution brain SPECT imaging.” *J Neuropsychiatry Clin Neurosci* **21**(4) 430–439

Audenaert, K, Van Laere, K, Dumont, F, Slegers, G, Mertens, J, van Heeringen, C, Dierckx, RA, 2001, “Decreased frontal serotonin 5-HT 2a receptor binding index in deliberate self-harm patients.” *Eur J Nucl Med* **28**(2) 175–182

Audenaert, K, Goethals, I, Van Laere, K, Lahorte, P, Brans, B, Versijpt, J, Vervaet, M, Beelaert, L, Van Heeringen, K, Dierckx, R, 2002, “SPECT neuropsychological activation procedure with the Verbal Fluency Test in attempted suicide patients” *Nucl Med Commun* **23**(9) 907–916

Baldacara, L, Nery-Fernandes, F, Rocha, M, Quarantini, LC, Rocha, GG, Guimaraes, JL, Araujo, C, Oliveira, I, Miranda-Scippa, A, Jackowski, A, 2011, “Is cerebellar volume related to bipolar disorder?” *J Affect Disord* **135**(1-3) 305–309

Ballard, ED, Lally, N, Nugent, AC, Furey, ML, Luckenbaugh, DA, Zarate, CA, 2014, “Neural correlates of suicidal ideation and its reduction in depression.” *Int J Neuropsychopharmacol* **18**(1)

Benedetti, F, Radaelli, D, Poletti, S, Locatelli, C, Falini, A, Colombo, C, Smeraldi, E, 2011, “Opposite effects of suicidality and lithium on gray matter volumes in bipolar depression.” *J Affect Disord* **135**(1-3) 139–147

Benedetti, F, Riccaboni, R, Poletti, S, Radaelli, D, Locatelli, C, Lorenzi, C, Pirovano, A, Smeraldi, E, Colombo, C, 2014, “The serotonin transporter genotype modulates the relationship between early stress and adult suicidality in bipolar disorder.” *Bipolar Disord* **16**(8) 857–866

Besteher, B, Wagner, G, Koch, K, Schachtzabel, C, Reichenbach, JR, Schlösser, R, Sauer, H, Schultz, CC, 2016, “Pronounced prefronto-temporal cortical thinning in schizophrenia: Neuroanatomical correlate of suicidal behavior” *Schizophr Res* **176**(2-3) 151–157

Bijttebier, S, Caeyenberghs, K, van den Ameele, H, Achten, E, Rujescu, D, Titeca, K, van Heeringen, C, 2015, “The Vulnerability to Suicidal Behavior is Associated with Reduced Connectivity Strength.” *Front Hum Neurosci* **9**(632

Budisic, M, Mislav, B, Karlovic, D, Dalibor, K, Trkanjec, Z, Zlatko, T, Lovrencic-Huzjan, A, Arijana, LH, Vukovic, V, Vlasta, V, Bosnjak, J, Jelena, B, Demarin, V, Vida, D, 2010, “Brainstem raphe lesion in patients with major depressive disorder and in patients with suicidal ideation recorded on transcranial sonography.” *Eur Arch Psychiatry Clin Neurosci* **260**(3) 203–208

Cannon, DM, Ichise, M, Fromm, SJ, Nugent, AC, Rollis, D, Gandhi, SK, Klaver, JM, Charney, DS, Manji, HK, Drevets, WC, 2006, “Serotonin transporter binding in bipolar disorder assessed using [11C]DASB and positron emission tomography” *Biol Psychiatry* **60**(3) 207–217

Cao, J, Chen, JM, Kuang, L, Ai, M, Fang, WD, Gan, Y, Wang, W, Chen, XR, Xu, XM, Wang, HG, Lv, Z, 2015, “Abnormal regional homogeneity in young adult suicide attempters with no diagnosable psychiatric disorder: a resting state functional magnetic imaging study.” *Psychiatry Res* **231**(2) 95–102

Cao, J, Chen, X, Chen, J, Ai, M, Gan, Y, Wang, W, Lv, Z, Zhang, S, Zhang, S, Wang, S, Kuang, L, Fang, W, 2016, “Resting-state functional MRI of abnormal baseline brain activity in young depressed patients with and without suicidal behavior.” *J Affect Disord* **205**(252–263

Chase, HW, Segreti, AM, Keller, TA, Cherkassky, VL, Just, MA, Pan, LA, Brent, DA, 2017, “Alterations of functional connectivity and intrinsic activity within the cingulate cortex of suicidal ideators.” *J Affect Disord* **212**(78–85

Chen, Z, Zhang, H, Jia, Z, Zhong, J, Huang, X, Du, M, Chen, L, Kuang, W, Sweeney, JA, Gong, Q, 2015, “Magnetization transfer imaging of suicidal patients with major depressive disorder.” *Sci Rep* **5**(9670

Colle, R, Chupin, M, Cury, C, Vandendrie, C, Gressier, F, Hardy, P, Falissard, B, Colliot, O, Ducreux, D, Corruble, E, 2015, “Depressed suicide attempters have smaller hippocampus than depressed patients without suicide attempts.” *J Psychiatr Res* **61**(13–18

Cox Lippard, ET, Johnston, JA, Blumberg, HP, 2014, “Neurobiological risk factors for suicide: insights from brain imaging.” *Am J Prev Med* **47**(3 Suppl 2) S152–62

Cullen, KR, Westlund, MK, Klimes-Dougan, B, Mueller, BA, Houri, A, Eberly, LE, Lim, KO, 2014, “Abnormal amygdala resting-state functional connectivity in adolescent depression.” *JAMA Psychiatry* **71**(10) 1138–1147

Cyprien, F, Courtet, P, Malafosse, A, Maller, J, Meslin, C, Bonafe, A, Le Bars, E, de Champfleur, NM, Ritchie, K, Artero, S, 2011, “Suicidal behavior is associated with reduced corpus callosum area.” *Biol Psychiatry* **70**(4) 320–326

Cyprien, F, de Champfleur, NM, Deverdun, J, Olié, E, Le Bars, E, Bonafé, A, Mura, T, Jollant, F, Courtet, P, Artero, S, 2016, “Corpus callosum integrity is affected by mood disorders and also by the suicide attempt history: A diffusion tensor imaging study.” *J Affect Disord* **206**(115–124

Desmyter, S, van Heeringen, C, Audenaert, K, 2011, “Structural and functional neuroimaging studies of the suicidal brain.” *Prog Neuropsychopharmacol Biol Psychiatry* **35**(4) 796–808

Ding, Y, Lawrence, N, Olie, E, Cyprien, F, le Bars, E, Bonafe, A, Phillips, ML, Courtet, P, Jollant, F, 2015, “Prefrontal cortex markers of suicidal vulnerability in mood disorders: a model-based structural neuroimaging study with a translational perspective.” *Transl Psychiatry* **5**(e516

Ding, Y, Pereira, F, Hoehne, A, Beaulieu, MM, Lepage, M, Turecki, G, Jollant, F, 2016, “Altered brain processing of decision-making in healthy first-degree biological relatives of suicide completers.” *Mol Psychiatry* (

Dombrovski, AY, Siegle, GJ, Szanto, K, Clark, L, Reynolds, CF, Aizenstein, H, 2012, “The temptation of suicide: striatal gray matter, discounting of delayed rewards, and suicide attempts in late-life depression.” *Psychol Med* **42**(6) 1203–1215

Dombrovski, AY, Szanto, K, Clark, L, Reynolds, CF, Siegle, GJ, 2013, “Reward Signals, Attempted Suicide, and Impulsivity in Late-Life Depression.” *JAMA Psychiatry* **70**(10) 1020–1030

Du, L, Zeng, J, Liu, H, Tang, D, Meng, H, Li, Y, Fu, Y, 2017, “Fronto-limbic disconnection in depressed patients with suicidal ideation: A resting-state functional connectivity study.” *J Affect Disord* **215**(213–217

Duarte, DGG, Neves, MCL, Albuquerque, MR, Turecki, G, Ding, Y, de Souza-Duran, FL, Busatto, G, Correa, H, 2017, “Structural brain abnormalities in patients with type I bipolar disorder and suicidal behavior.” *Psychiatry Res* **265**(9–17

Ehrlich, S, Breeze, JL, Hesdorffer, DC, Noam, GG, Hong, X, Alban, RL, Davis, SE, Renshaw, PF, 2005, “White matter hyperintensities and their association with suicidality in depressed young adults” *J Affect Disord* **86**(2-3) 281–287

Ehrlich, S, Noam, GG, Lyoo, IK, Kwon, BJ, Clark, MA, Renshaw, PF, 2003, “Subanalysis of the location of white matter hyperintensities and their association with suicidality in children and youth” *Ann N Y Acad Sci* **1008**(265–268

Ehrlich, S, Noam, GG, Lyoo, IK, Kwon, BJ, Clark, MA, Renshaw, PF, 2004, “White matter hyperintensities and their associations with suicidality in psychiatrically hospitalized children and adolescents” *J Am Acad Child Adolesc Psychiatry* **43**(6) 770–776

Fan, T, Wu, X, Yao, L, Dong, J, 2013, “Abnormal baseline brain activity in suicidal and non-suicidal patients with major depressive disorder.” *Neurosci Lett* **534**(35–40

Fountoulakis, KN, Iacovides, A, Fotiou, F, Nimatoudis, J, Bascialla, F, Ioannidou, C, Kaprinis, G, Bech, P, 2004, “Neurobiological and psychological correlates of suicidal attempts and thoughts of death in patients with major depression” *Neuropsychobiology* **49**(1) 42–52

Fradkin, Y, Khadka, S, Bessette, KL, Stevens, MC, 2016, “The relationship of impulsivity and cortical thickness in depressed and non-depressed adolescents.” *Brain Imaging Behav* (

Giakoumatos, CI, Tandon, N, Shah, J, Mathew, IT, Brady, RO, Clementz, BA, Pearlson, GD, Thaker, GK, Tamminga, CA, Sweeney, JA, Keshavan, MS, 2013, “Are structural brain abnormalities associated with suicidal behavior in patients with psychotic disorders?” *J Psychiatr Res* **47**(10) 1389–1395

Gifuni, AJ, Ding, Y, Olie, E, Lawrence, N, Cyprien, F, Le Bars, E, Bonafe, A, Phillips, ML, Courtet, P, Jollant, F, 2016, “Subcortical nuclei volumes in suicidal behavior: nucleus accumbens may modulate the lethality of acts.” *Brain Imaging Behav* **10**(1) 96–104

Gifuni, AJ, Olié, E, Ding, Y, Cyprien, F, le Bars, E, Bonafé, A, Courtet, P, Jollant, F, 2017, “Corpus callosum volumes in bipolar disorders and suicidal vulnerability.” *Psychiatry Res* **262**(47–54

Goodman, M, Hazlett, EA, Avedon, JB, Siever, DR, Chu, KW, New, AS, 2011, “Anterior cingulate volume reduction in adolescents with borderline personality disorder and co-morbid major depression.” *J Psychiatr Res* **45**(6) 803–807

Gosnell, SN, Velasquez, KM, Molfese, DL, Molfese, PJ, Madan, A, Fowler, JC, Christopher Frueh, B, Baldwin, PR, Salas, R, 2016, “Prefrontal cortex, temporal cortex, and hippocampus volume are affected in suicidal psychiatric patients.” *Psychiatry Res* **256**(50–56

Grangeon, MC, Seixas, C, Quarantini, LC, Miranda-Scippa, A, Pompili, M, Steffens, DC, Wenzel, A, Lacerda, AL, de Oliveira, IR, 2010, “White matter hyperintensities and their association with suicidality in major affective disorders: a meta-analysis of magnetic resonance imaging studies.” *CNS Spectr* **15**(6) 375–381

Harenski, CL, Brook, M, Kosson, DS, Bustillo, JR, Harenski, KA, Caldwell, MF, Van Rybroek, GJ, Koenigs, M, Decety, J, Thornton, DM, Calhoun, VD, Kiehl, KA, 2017, “Socio-neuro risk factors for suicidal behavior in criminal offenders with psychotic disorders.” *Soc Cogn Affect Neurosci* (

Hwang, JP, Lee, TW, Tsai, SJ, Chen, TJ, Yang, CH, Lirng, JF, Tsai, CF, 2010, “Cortical and subcortical abnormalities in late-onset depression with history of suicide attempts investigated with MRI and voxel-based morphometry.” *J Geriatr Psychiatry Neurol* **23**(3) 171–184

Jia, Z, Huang, X, Wu, Q, Zhang, T, Lui, S, Zhang, J, Amatya, N, Kuang, W, Chan, RC, Kemp, GJ, Mechelli, A, Gong, Q, 2010, “High-Field Magnetic Resonance Imaging of Suicidality in Patients With Major Depressive Disorder.” *Am J Psychiatry* **67**(11) 1381–1390

Jia, Z, Wang, Y, Huang, X, Kuang, W, Wu, Q, Lui, S, Sweeney, JA, Gong, Q, 2014, “Impaired frontothalamic circuitry in suicidal patients with depression revealed by diffusion tensor imaging at 3.0 T.” *J Psychiatry Neurosci* **39**(3) 170–177

Johnston, JA, Wang, F, Liu, J, Blond, BN, Wallace, A, Liu, J, Spencer, L, Cox Lippard, ET, Purves, KL, Landeros-Weisenberger, A, Hermes, E, Pittman, B, Zhang, S, King, R, Martin, A, Oquendo, MA, Blumberg, HP, 2017, “Multimodal Neuroimaging of Frontolimbic Structure and Function Associated With Suicide Attempts in Adolescents and Young Adults With Bipolar Disorder.” *Am J Psychiatry* (appiajp201615050652

Jollant, F, Lawrence, NL, Olie, E, Guillaume, S, Courtet, P, 2011, “The suicidal mind and brain: A review of neuropsychological and neuroimaging studies.” *World J Biol Psychiatry* **12**(5) 319–339

Jollant, F, Lawrence, NS, Giampietro, V, Brammer, MJ, Fullana, MA, Drapier, D, Courtet, P, Phillips, ML, 2008, “Orbitofrontal cortex response to angry faces in men with histories of suicide attempts.” *Am J Psychiatry* **165**(6) 740–748

Jollant, F, Lawrence, NS, Olie, E, O’Daly, O, Malafosse, A, Courtet, P, Phillips, ML, 2010, “Decreased activation of lateral orbitofrontal cortex during risky choices under uncertainty is associated with disadvantageous decision-making and suicidal behavior.” *Neuroimage* **51**(3) 1275–1281

Jollant, F, Near, J, Turecki, G, Richard-Devantoy, S, 2017, “Spectroscopy markers of suicidal risk and mental pain in depressed patients.” *Prog Neuropsychopharmacol Biol Psychiatry* **73**(64–71

Jovev, M, Garner, B, Phillips, L, Velakoulis, D, Wood, SJ, Jackson, HJ, Pantelis, C, McGorry, PD, Chanen, AM, 2008, “An MRI study of pituitary volume and parasuicidal behavior in teenagers with first-presentation borderline personality disorder” *Psychiatry Res* **162**(3) 273–277

Kang, SG, Na, KS, Choi, JW, Kim, JH, Son, YD, Lee, YJ, 2017, “Resting-state functional connectivity of the amygdala in suicide attempters with major depressive disorder.” *Prog Neuropsychopharmacol Biol Psychiatry* (

Kim, B, Oh, J, Kim, MK, Lee, S, Tae, WS, Kim, CM, Choi, TK, Lee, SH, 2015, “White matter alterations are associated with suicide attempt in patients with panic disorder.” *J Affect Disord* **175**(139–146

Kim, YJ, Park, HJ, Jahng, GH, Lee, SM, Kang, WS, Kim, SK, Kim, T, Cho, AR, Park, JK, 2017, “A pilot study of differential brain activation to suicidal means and DNA methylation of CACNA1C gene in suicidal attempt patients.” *Psychiatry Res* **255**(42–48

Kolla, NJ, Chiuccariello, L, Wilson, AA, Houle, S, Links, P, Bagby, RM, McMain, S, Kellow, C, Patel, J, Rekkas, PV, Pasricha, S, Meyer, JH, 2016, “Elevated Monoamine Oxidase-A Distribution Volume in Borderline Personality Disorder Is Associated With Severity Across Mood Symptoms, Suicidality, and Cognition.” *Biol Psychiatry* **79**(2) 117–126

Lee, SJ, Kim, B, Oh, D, Kim, MK, Kim, KH, Bang, SY, Choi, TK, Lee, SH, 2016a, “White matter alterations associated with suicide in patients with schizophrenia or schizophreniform disorder.” *Psychiatry Res* **248**(23–29

Lee, YJ, Kim, S, Gwak, AR, Kim, SJ, Kang, SG, Na, KS, Son, YD, Park, J, 2016b, “Decreased regional gray matter volume in suicide attempters compared to suicide non-attempters with major depressive disorders.” *Compr Psychiatry* **67**(59–65

Leyton, M, Paquette, V, Gravel, P, Rosa-Neto, P, Weston, F, Diksic, M, Benkelfat, C, 2006, “alpha-[11C]Methyl-L-tryptophan trapping in the orbital and ventral medial prefrontal cortex of suicide attempters” *Eur Neuropsychopharmacol* **16**(3) 220–223

Lijffijt, M, Rourke, ED, Swann, AC, Zunta-Soares, GB, Soares, JC, 2014, “Illness-course modulates suicidality-related prefrontal gray matter reduction in women with bipolar disorder.” *Acta Psychiatr Scand* **130**(5) 374–387

Lindstrom, MB, Ryding, E, Bosson, P, Ahnlide, JA, Rosen, I, Traskman-Bendz, L, 2004, “Impulsivity related to brain serotonin transporter binding capacity in suicide attempters” *Eur Neuropsychopharmacol* **14**(4) 295–300

Lischke, A, Domin, M, Freyberger, HJ, Grabe, HJ, Mentel, R, Bernheim, D, Lotze, M, 2017, “Structural Alterations in the Corpus Callosum Are Associated with Suicidal Behavior in Women with Borderline Personality Disorder.” *Front Hum Neurosci* **11**(196

Mahon, K, Burdick, KE, Wu, J, Ardekani, BA, Szeszko, PR, 2012, “Relationship between suicidality and impulsivity in bipolar I disorder: a diffusion tensor imaging study.” *Bipolar Disord* **14**(1) 80–89

Marchand, WR, Lee, JN, Garn, C, Thatcher, J, Gale, P, Kreitschitz, S, Johnson, S, Wood, N, 2011, “Striatal and cortical midline activation and connectivity associated with suicidal ideation and depression in bipolar II disorder.” *J Affect Disord* **133**(3) 638–645

Marchand, WR, Lee, JN, Johnson, S, Gale, P, Thatcher, J, 2013a, “Differences in functional connectivity in major depression versus bipolar II depression.” *J Affect Disord* **150**(2) 527–532

Marchand, WR, Lee, JN, Johnson, S, Thatcher, J, Gale, P, 2013b, “Striatal circuit function is associated with prior self-harm in remitted major depression.” *Neurosci Lett* (

Marchand, WR, Lee, JN, Johnson, S, Thatcher, J, Gale, P, Wood, N, Jeong, EK, 2012, “Striatal and cortical midline circuits in major depression: implications for suicide and symptom expression.” *Prog Neuropsychopharmacol Biol Psychiatry* **36**(2) 290–299

Martin, PC, Zimmer, TJ, Pan, LA, 2015, “Magnetic resonance imaging markers of suicide attempt and suicide risk in adolescents.” *CNS Spectr* **20**(4) 355–358

Matsuo, K, Nielsen, N, Nicoletti, MA, Hatch, JP, Monkul, ES, Watanabe, Y, Zunta-Soares, GB, Nery, FG, Soares, JC, 2010, “Anterior genu corpus callosum and impulsivity in suicidal patients with bipolar disorder.” *Neurosci Lett* **469**(1) 75–80

Meyer, JH, McMain, S, Kennedy, SH, Korman, L, Brown, GM, DaSilva, JN, Wilson, AA, Blak, T, Eynan-Harvey, R, Goulding, VS, Houle, S, Links, P, 2003, “Dysfunctional attitudes and 5-HT2 receptors during depression and self-harm” *Am J Psychiatry* **160**(1) 90–99

Miller, JM, Everett, BA, Oquendo, MA, Ogden, RT, Mann, JJ, Parsey, RV, 2016, “Positron emission tomography quantification of serotonin transporter binding in medication-free bipolar disorder.” *Synapse* **70**(1) 24–32

Miller, JM, Hesselgrave, N, Ogden, RT, Sullivan, GM, Oquendo, MA, Mann, JJ, Parsey, RV, 2013, “Positron emission tomography quantification of serotonin transporter in suicide attempters with major depressive disorder.” *Biol Psychiatry* **74**(4) 287–295

Minzenberg, MJ, Lesh, T, Niendam, T, Yoon, JH, Cheng, Y, Rhoades, R, Carter, CS, 2015a, “Conflict-related anterior cingulate functional connectivity is associated with past suicidal ideation and behavior in recent-onset schizophrenia.” *J Psychiatr Res* **65**(95–101

Minzenberg, MJ, Lesh, T, Niendam, T, Yoon, JH, Cheng, Y, Rhoades, RN, Carter, CS, 2015b, “Frontal Motor Cortex Activity During Reactive Control Is Associated With Past Suicidal Behavior in Recent-Onset Schizophrenia.” *Crisis* **36**(5) 363–370

Minzenberg, MJ, Lesh, TA, Niendam, TA, Cheng, Y, Carter, CS, 2016, “Conflict-Related Anterior Cingulate Functional Connectivity Is Associated With Past Suicidal Ideation and Behavior in Recent-Onset Psychotic Major Mood Disorders.” *J Neuropsychiatry Clin Neurosci* (appineuropsych15120422

Minzenberg, MJ, Lesh, TA, Niendam, TA, Yoon, JH, Cheng, Y, Rhoades, RN, Carter, CS, 2015c, “Control-related frontal-striatal function is associated with past suicidal ideation and behavior in patients with recent-onset psychotic major mood disorders.” *J Affect Disord* **188**(202–209

Minzenberg, MJ, Lesh, TA, Niendam, TA, Yoon, JH, Rhoades, RN, Carter, CS, 2014, “Frontal cortex control dysfunction related to long-term suicide risk in recent-onset schizophrenia.” *Schizophr Res* **157**(1-3) 19–25

Monkul, ES, Hatch, JP, Nicoletti, MA, Spence, S, Brambilla, P, Lacerda, AL, Sassi, RB, Mallinger, AG, Keshavan, MS, Soares, JC, 2007, “Fronto-limbic brain structures in suicidal and non-suicidal female patients with major depressive disorder” *Mol Psychiatry* **12**(4) 360–366

Myung, W, Han, CE, Fava, M, Mischoulon, D, Papakostas, GI, Heo, JY, Kim, KW, Kim, ST, Kim, DJ, Kim, DK, Seo, SW, Seong, JK, Jeon, HJ, 2016, “Reduced frontal-subcortical white matter connectivity in association with suicidal ideation in major depressive disorder.” *Transl Psychiatry* **6**(e835

Nery-Fernandes, F, Rocha, MV, Jackowski, A, Ladeia, G, Guimaraes, JL, Quarantini, LC, Araujo-Neto, CA, De Oliveira, IR, Miranda-Scippa, A, 2012, “Reduced posterior corpus callosum area in suicidal and non-suicidal patients with bipolar disorder.” *J Affect Disord* **142**(1-3) 150–155

Nye, JA, Purselle, D, Plisson, C, Voll, RJ, Stehouwer, JS, Votaw, JR, Kilts, CD, Goodman, MM, Nemeroff, CB, 2013, “Decreased brainstem and putamen SERT binding potential in depressed suicide attempters using [ C]-ZIENT PET imaging.” *Depress Anxiety* **30**(10) 902–907

Olié, E, Ding, Y, Le Bars, E, de Champfleur, NM, Mura, T, Bonafé, A, Courtet, P, Jollant, F, 2015, “Processing of decision-making and social threat in patients with history of suicidal attempt: A neuroimaging replication study.” *Psychiatry Res* (

Olié, E, Jollant, F, Deverdun, J, de Champfleur, NM, Cyprien, F, Le Bars, E, Mura, T, Bonafé, A, Courtet, P, 2017, “The experience of social exclusion in women with a history of suicidal acts: a neuroimaging study.” *Sci Rep* **7**(1) 89

Olvet, DM, Peruzzo, D, Thapa-Chhetry, B, Sublette, ME, Sullivan, GM, Oquendo, MA, Mann, JJ, Parsey, RV, 2014, “A diffusion tensor imaging study of suicide attempters.” *J Psychiatr Res* (

Oquendo, MA, Hastings, RS, Huang, YY, Simpson, N, Ogden, RT, Hu, XZ, Goldman, D, Arango, V, Van Heertum, RL, Mann, JJ, Parsey, RV, 2007, “Brain serotonin transporter binding in depressed patients with bipolar disorder using positron emission tomography” *Arch Gen Psychiatry* **64**(2) 201–208

Oquendo, MA, Placidi, GP, Malone, KM, Campbell, C, Keilp, J, Brodsky, B, Kegeles, LS, Cooper, TB, Parsey, RV, van Heertum, RL, Mann, JJ, 2003, “Positron emission tomography of regional brain metabolic responses to a serotonergic challenge and lethality of suicide attempts in major depression” *Arch Gen Psychiatry* **60**(1) 14–22

Oquendo, MA, Galfalvy, H, Sullivan, GM, Miller, JM, Milak, MM, Sublette, ME, Cisneros-Trujillo, S, Burke, AK, Parsey, RV, Mann, JJ, 2016, “Positron Emission Tomographic Imaging of the Serotonergic System and Prediction of Risk and Lethality of Future Suicidal Behavior.” *JAMA Psychiatry* **73**(10) 1048–1055

Pan, L, Segreti, A, Almeida, J, Jollant, F, Lawrence, N, Brent, D, Phillips, M, 2013a, “Preserved hippocampal function during learning in the context of risk in adolescent suicide attempt.” *Psychiatry Res* **211**(2) 112–118

Pan, LA, Batezati-Alves, SC, Almeida, JR, Segreti, A, Akkal, D, Hassel, S, Lakdawala, S, Brent, DA, Phillips, ML, 2011, “Dissociable patterns of neural activity during response inhibition in depressed adolescents with and without suicidal behavior.” *J Am Acad Child Adolesc Psychiatry* **50**(6) 602–611.e3

Pan, LA, Hassel, S, Segreti, AM, Nau, SA, Brent, DA, Phillips, ML, 2013b, “Differential patterns of activity and functional connectivity in emotion processing neural circuitry to angry and happy faces in adolescents with and without suicide attempt.” *Psychol Med* **43**(10) 2129–2142

Pan, LA, Ramos, L, Segreti, A, Brent, DA, Phillips, ML, 2015, “Right superior temporal gyrus volume in adolescents with a history of suicide attempt.” *Br J Psychiatry* **206**(4) 339–340

Parsey, RV, Hastings, RS, Oquendo, MA, Huang, YY, Simpson, N, Arcement, J, Huang, Y, Ogden, RT, Van Heertum, RL, Arango, V, Mann, JJ, 2006, “Lower serotonin transporter binding potential in the human brain during major depressive episodes” *Am J Psychiatry* **163**(1) 52–58

Peng, H, Wu, K, Li, J, Qi, H, Guo, S, Chi, M, Wu, X, Guo, Y, Yang, Y, Ning, Y, 2014, “Increased suicide attempts in young depressed patients with abnormal temporal-parietal-limbic gray matter volume.” *J Affect Disord* **165**(69–73

Pompili, M, Ehrlich, S, De Pisa, E, Mann, JJ, Innamorati, M, Cittadini, A, Montagna, B, Iliceto, P, Romano, A, Amore, M, Tatarelli, R, Girardi, P, 2007, “White matter hyperintensities and their associations with suicidality in patients with major affective disorders” *Eur Arch Psychiatry Clin Neurosci* **257**(8) 494–499

Pompili, M, Innamorati, M, Mann, JJ, Oquendo, MA, Lester, D, Del Casale, A, Serafini, G, Rigucci, S, Romano, A, Tamburello, A, Manfredi, G, De Pisa, E, Ehrlich, S, Giupponi, G, Amore, M, Tatarelli, R, Girardi, P, 2008, “Periventricular white matter hyperintensities as predictors of suicide attempts in bipolar disorders and unipolar depression” *Prog Neuropsychopharmacol Biol Psychiatry* **32**(6) 1501–1507

Pu, S, Nakagome, K, Yamada, T, Yokoyama, K, Matsumura, H, Yamada, S, Sugie, T, Miura, A, Mitani, H, Iwata, M, Nagata, I, Kaneko, K, 2015, “Suicidal ideation is associated with reduced prefrontal activation during a verbal fluency task in patients with major depressive disorder.” *J Affect Disord* **181**(9–17

Quevedo, K, Ng, R, Scott, H, Martin, J, Smyda, G, Keener, M, Oppenheimer, CW, 2016, “The neurobiology of self-face recognition in depressed adolescents with low or high suicidality.” *J Abnorm Psychol* **125**(8) 1185–1200

Reisch, T, Seifritz, E, Esposito, F, Wiest, R, Valach, L, Michel, K, 2010, “An fMRI study on mental pain and suicidal behavior.” *J Affect Disord* **126**(1-2) 321–5.

Richard-Devantoy, S, Ding, Y, Lepage, M, Turecki, G, Jollant, F, 2016a, “Cognitive inhibition in depression and suicidal behavior: a neuroimaging study.” *Psychol Med* **46**(5) 933–944

Richard-Devantoy, S, Turecki, G, Jollant, F, 2016b, “Neurobiology of Elderly Suicide.” *Arch Suicide Res* (

Rocha, MV, Nery-Fernandes, F, Guimarães, JL, Quarantini, LC, de Oliveira, IR, Ladeia-Rocha, GG, Jackowski, AP, de Araujo Neto, C, Miranda-Scippa, Â, 2015, “Normal Metabolic Levels in Prefrontal Cortex in Euthymic Bipolar I Patients with and without Suicide Attempts.” *Neural Plast* **2015**(165180

Rusch, N, Spoletini, I, Wilke, M, Martinotti, G, Bria, P, Trequattrini, A, Bonaviri, G, Caltagirone, C, Spalletta, G, 2008, “Inferior frontal white matter volume and suicidality in schizophrenia” *Psychiatry Res* **164**(3) 206–214

Ryding, E, Ahnlide, JA, Lindstrom, M, Rosen, I, Traskman-Bendz, L, 2006, “Regional brain serotonin and dopamine transporter binding capacity in suicide attempters relate to impulsiveness and mental energy” *Psychiatry Res* **148**(2-3) 195–203

Sachs-Ericsson, N, Hames, JL, Joiner, TE, Corsentino, E, Rushing, NC, Palmer, E, Gotlib, IH, Selby, EA, Zarit, S, Steffens, DC, 2014, “Differences between suicide attempters and nonattempters in depressed older patients: depression severity, white-matter lesions, and cognitive functioning.” *Am J Geriatr Psychiatry* **22**(1) 75–85

Sobanski, T, Bar, KJ, Wagner, G, 2015, “Neural, cognitive, and neuroimaging markers of the suicidal brain” *Report in Med maging* **8**(71–81

Soloff, P, White, R, Diwadkar, VA, 2014a, “Impulsivity, aggression and brain structure in high and low lethality suicide attempters with borderline personality disorder.” *Psychiatry Res* **222**(3) 131–139

Soloff, PH, Chiappetta, L, Mason, NS, Becker, C, Price, JC, 2014b, “Effects of serotonin-2A receptor binding and gender on personality traits and suicidal behavior in borderline personality disorder.” *Psychiatry Res* **222**(3) 140–148

Soloff, PH, Price, JC, Meltzer, CC, Fabio, A, Frank, GK, Kaye, WH, 2007, “5HT2A receptor binding is increased in borderline personality disorder.” *Biol Psychiatry* **62**(6) 580–587

Soloff, PH, Pruitt, P, Sharma, M, Radwan, J, White, R, Diwadkar, VA, 2012, “Structural brain abnormalities and suicidal behavior in borderline personality disorder.” *J Psychiatr Res* (

Spoletini, I, Piras, F, Fagioli, S, Rubino, IA, Martinotti, G, Siracusano, A, Caltagirone, C, Spalletta, G, 2011, “Suicidal attempts and increased right amygdala volume in schizophrenia.” *Schizophr Res* **125**(1) 30–40

Sublette, ME, Milak, MS, Galfalvy, HC, Oquendo, MA, Malone, KM, Mann, JJ, 2013, “Regional brain glucose uptake distinguishes suicide attempters from non-attempters in major depression.” *Arch Suicide Res* **17**(4) 434–447

Sullivan, GM, Oquendo, MA, Milak, M, Miller, JM, Burke, A, Ogden, RT, Parsey, RV, Mann, JJ, 2015, “Positron emission tomography quantification of serotonin(1A) receptor binding in suicide attempters with major depressive disorder.” *JAMA Psychiatry* **72**(2) 169–178

Tsujii, N, Mikawa, W, Tsujimoto, E, Adachi, T, Niwa, A, Ono, H, Shirakawa, O, 2017, “Reduced left precentral regional responses in patients with major depressive disorder and history of suicide attempts.” *PLoS One* **12**(4) e0175249

van Heeringen, C, Audenaert, K, Van Laere, K, Dumont, F, Slegers, G, Mertens, J, Dierckx, RA, 2003, “Prefrontal 5-HT2a receptor binding index, hopelessness and personality characteristics in attempted suicide.” *J Affect Disord* **74**(2) 149–158

van Heeringen, K, Bijttebier, S, Desmyter, S, Vervaet, M, Baeken, C, 2014, “Is there a neuroanatomical basis of the vulnerability to suicidal behavior? A coordinate-based meta-analysis of structural and functional MRI studies.” *Front Hum Neurosci* **8**(824

van Heeringen, K, Wu, GR, Vervaet, M, Vanderhasselt, MA, Baeken, C, 2017, “Decreased resting state metabolic activity in frontopolar and parietal brain regions is associated with suicide plans in depressed individuals.” *J Psychiatr Res* **84**(243–248

Vang, FJ, Ryding, E, Traskman-Bendz, L, van Westen, D, Lindstrom, MB, 2010, “Size of basal ganglia in suicide attempters, and its association with temperament and serotonin transporter density.” *Psychiatry Res* **183**(2) 177–179

Vanyukov, PM, Szanto, K, Hallquist, MN, Siegle, GJ, Reynolds, CF, Forman, SD, Aizenstein, HJ, Dombrovski, AY, 2016, “Paralimbic and lateral prefrontal encoding of reward value during intertemporal choice in attempted suicide.” *Psychol Med* **46**(2) 381–391

Vanyukov, PM, Szanto, K, Siegle, GJ, Hallquist, MN, Reynolds, CF, Aizenstein, HJ, Dombrovski, AY, 2015, “Impulsive Traits and Unplanned Suicide Attempts Predict Exaggerated Prefrontal Response to Angry Faces in the Elderly.” *Am J Geriatr Psychiatry* **23**(8) 829–839

Wagner, G, Koch, K, Schachtzabel, C, Schultz, CC, Sauer, H, Schlosser, RG, 2011, “Structural brain alterations in patients with major depressive disorder and high risk for suicide: evidence for a distinct neurobiological entity?” *Neuroimage* **54**(2) 1607–1614

Wagner, G, Schultz, CC, Koch, K, Schachtzabel, C, Sauer, H, Schlosser, RG, 2012, “Prefrontal cortical thickness in depressed patients with high-risk for suicidal behavior.” *J Psychiatr Res* **46**(11) 1449–1455

Willeumier, K, Taylor, DV, Amen, DG, 2011, “Decreased cerebral blood flow in the limbic and prefrontal cortex using SPECT imaging in a cohort of completed suicides.” *Transl Psychiatry* **1**(e28

Yeh, YW, Ho, PS, Chen, CY, Kuo, SC, Liang, CS, Ma, KH, Shiue, CY, Huang, WS, Cheng, CY, Wang, TY, Lu, RB, Huang, SY, 2014, “Incongruent reduction of serotonin transporter associated with suicide attempts in patients with major depressive disorder: a positron emission tomography study with 4-[18F]-ADAM.” *Int J Neuropsychopharmacol* **18**(3)

Yeh, YW, Ho, PS, Chen, CY, Kuo, SC, Liang, CS, Yen, CH, Huang, CC, Shiue, CY, Huang, WS, Ma, KH, Lu, RB, Huang, SY, 2015, “Suicidal ideation modulates the reduction in serotonin transporter availability in male military conscripts with major depression: A 4-[18F]-ADAM PET study.” *World J Biol Psychiatry* **16**(7) 502–512

Zhang, H, Chen, Z, Jia, Z, Gong, Q, 2014, “Dysfunction of neural circuitry in depressive patients with suicidal behaviors: a review of structural and functional neuroimaging studies.” *Prog Neuropsychopharmacol Biol Psychiatry* **53**(61–66

Zhang, H, Wei, X, Tao, H, Mwansisya, TE, Pu, W, He, Z, Hu, A, Xu, L, Liu, Z, Shan, B, Xue, Z, 2013, “Opposite effective connectivity in the posterior cingulate and medial prefrontal cortex between first-episode schizophrenic patients with suicide risk and healthy controls.” *PLoS One* **8**(5) e63477

Zhang, S, Chen, JM, Kuang, L, Cao, J, Zhang, H, Ai, M, Wang, W, Zhang, SD, Wang, SY, Liu, SJ, Fang, WD, 2016, “Association between abnormal default mode network activity and suicidality in depressed adolescents.” *BMC Psychiatry* **16**(1) 337

**Supplemental Figure. Decreased volumes in individuals with vs. without a family history of suicide in healthy individuals (N=17 vs. 16, respectively), patient controls (N=12 vs. 44), and suicide attempters (N=9 vs. 38) (from left to right; uncorrected level p<0.001).** The left (blue circle) and right (red circle) temporal clusters, and the right dorsolateral prefrontal cluster (green circle) are found across the three groups. Z coordinates are similar to Figure 2 in main text (left to right and top to down: -12, -1, 16, 38, and 49).


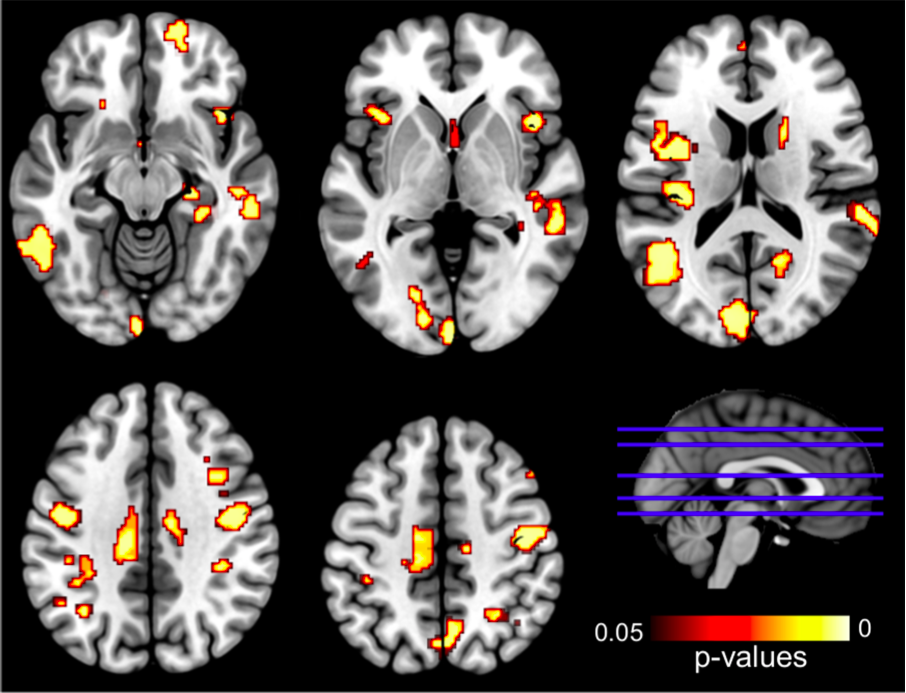

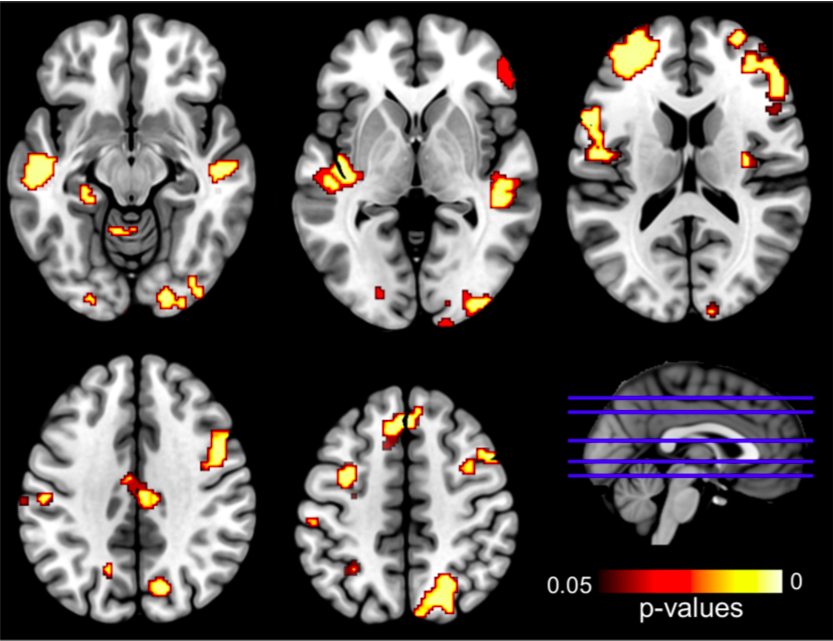

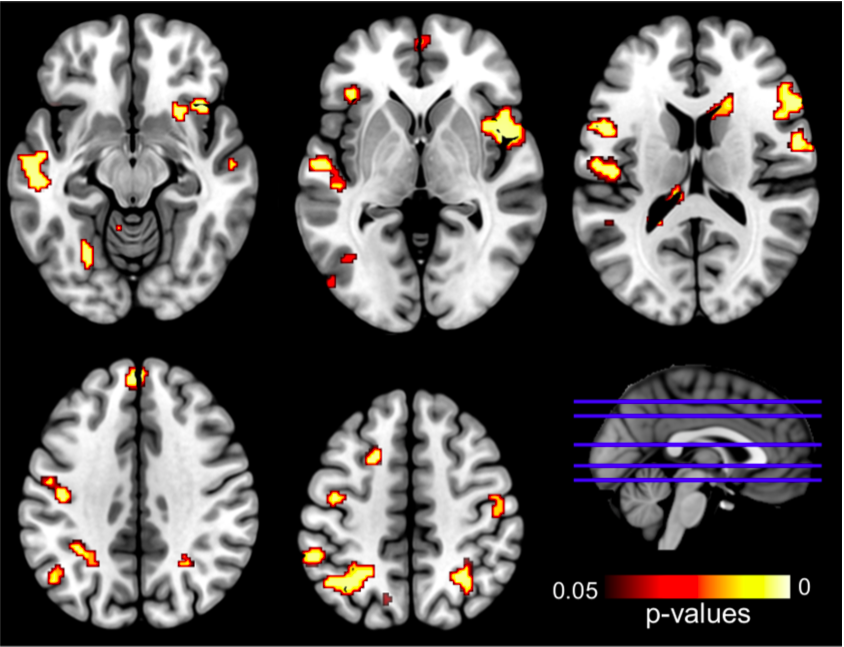

Supplement: Supplementary file 1 — Supplemental Material [file 41398_2018_170_MOESM1_ESM.docx]
